# Advances in 3D Bioprinting for Scaffold-Based and Scaffold-Free Tissue Engineering and Regenerative Medicine

**DOI:** 10.3390/gels12080691

**Published:** 2026-08-03

**Authors:** Kannan Badri Narayanan

**Affiliations:** 1 School of Chemical Engineering, Yeungnam University, 280 Daehak-Ro, Gyeongsan 38541, Gyeongbuk, Republic of Korea; okbadri@gmail.com or okbadri@yu.ac.kr; 2 Research Institute of Cell Culture, Yeungnam University, 280 Daehak-Ro, Gyeongsan 38541, Gyeongbuk, Republic of Korea

**Keywords:** 3D printing, extrusion-based, droplet-based, laser-mediated, light-based, scaffold-based, scaffold-free, bioassembly, tissue engineering, regenerative medicine

## Abstract

Three-dimensional (3D) bioprinting has emerged as a versatile biofabrication strategy that enables the precise, spatiotemporally controlled co-deposition of living cells, biomaterials, and bioactive agents, including growth factors, cytokines, and extracellular matrix (ECM) components, into geometrically defined 3D constructs. By translating digital design models derived from computed tomography (CT), magnetic resonance imaging (MRI), or computational modeling directly into physical tissue architectures, 3D bioprinting facilitates the assembly of hierarchically organized constructs that closely recapitulate the structural, mechanical, and functional characteristics of native tissues. The principal 3D bioprinting strategies are broadly classified into scaffold-based and scaffold-free approaches. Engineered bioinks, whether formulated as cell-laden natural, synthetic, or composite polymer hydrogels, tissue-derived decellularized ECM (dECM) components, or pure cellular spheroids and organoids, constitute the cornerstone of these biofabrication platforms. Scaffold-based 3D bioprinting comprises extrusion-based, droplet-based (inkjet and drop-on-demand), light-based vat photopolymerization (stereolithography and digital light processing), and laser-assisted bioprinting based on laser-induced forward transfer (LIFT). Each of these modalities imposes distinct constraints on bioink rheology, crosslinking mechanisms, spatial resolution, throughput, and post-printing cell viability; consequently, a specific 3D bioprinting strategy is selected according to the specific requirements of the target tissue application. Scaffold-free 3D bioprinting and bioassembly techniques, including the Kenzan method, aspiration-assisted bioprinting, magnetic bioprinting, and other field-directed tissue assembly approaches, enable the fabrication of spheroid- and organoid-based constructs without the necessity for exogenous biomaterial scaffolds. Because native tissues exhibit diversity in cellular composition, ECM architecture, mechanical properties, and physiological function, no individual bioprinting platform or bioink formulation serves as a universal 3D bioprinting solution. The engineering of biomimetic tissue constructs, therefore, requires the selection of application-tailored fabrication approaches. Under this biofabrication paradigm, 3D bioprinting has been applied across a wide range of tissue engineering targets, including skin, bone, cartilage, osteochondral interfaces, cardiac and vascular tissue, neural structures, ocular, dental, and adipose tissue. This review discusses recent advances in scaffold-based and scaffold-free 3D bioprinting applications for tissue engineering and regenerative medicine across diverse tissue systems.

## 1. Introduction

Additive manufacturing (AM), commonly known as three-dimensional (3D) printing, comprises a family of layer-by-layer (LbL) fabrication methods that deposit materials, including polymers, metals, ceramics, and composites, with spatial precision to reproduce complex geometries from computer-aided design (CAD) models. The origins of this technology trace its origins to Charles W. Hull, who developed the first stereolithography (SLA) apparatus and filed the patent application in August 1984 [1]. In this process, a focused ultraviolet beam selectively polymerizes a liquid photoresin in a vat, building objects LbL from the bottom upward. Initially described as “rapid prototyping,” this approach marked a paradigm shift from conventional subtractive manufacturing to the LbL construction of 3D objects. The term “3D printing” was subsequently trademarked in 1993 by Emanuel Sachs and colleagues at the Massachusetts Institute of Technology (MIT) to describe binder jetting, a powder-bed process in which a liquid binder is selectively deposited through inkjet print heads to fuse particles LbL [2,3]. The technology was later commercialized by Soligen Technologies, Extrude Hone Corporation, and Z Corporation, extending the reach of AM into industrial manufacturing. Subsequent developments yielded diverse AM techniques, including extrusion-based methods such as fused deposition modeling (FDM), direct ink writing (DIW), pellet extrusion, and semi-solid extrusion (SSE), as well as vat photopolymerization approaches such as SLA and digital light processing (DLP), and droplet-based inkjet systems.

AM of biomaterials has progressed from a rapid prototyping tool into a platform for patient-specific medical devices. Early in-human applications included polyetherketoneketone (PEKK) cranial bone plates for large cranial defect repair, customized from computed tomography (CT) and magnetic resonance imaging (MRI) data [4,5], and a bioresorbable PCL bronchial splint for the treatment of life-threatening tracheobronchomalacia in a pediatric patient. This splint was designed from CT imaging and fabricated by laser-based 3D printing, representing one of the first reported uses of a patient-specific, computer-aided, additively manufactured implant for airway reconstruction [6].

Tissue damage and degeneration remain leading contributors to global morbidity and mortality, with nearly 45% of deaths in the developed world directly attributable to pathological fibrosis, a destructive state of impaired tissue regeneration where healthy organ tissue is permanently replaced by scar tissue. To overcome this, the field of tissue engineering develops advanced biomaterial scaffolds and regenerative therapies designed to arrest fibrotic pathways and restore organ function [7,8,9]. The limited intrinsic regenerative capacity of many human tissues, including cartilage, myocardium, and neural tissue, emphasizes the urgent need for advanced strategies in tissue engineering and regenerative medicine. Traditional two-dimensional (2D) tissue culture platforms, such as tissue culture polystyrene (TCPS) substrates, have long supported in vitro studies of cellular processes, including proliferation, differentiation, and senescence. However, these planar systems fundamentally fail to recapitulate the physiological 3D architecture, cell–cell and cell–matrix interactions, mechanical cues, and biochemical gradients characteristic of native tissues; furthermore, they frequently distort cell morphology, gene expression, and functional behavior. To better mimic the in vivo microenvironment, tissue engineers have developed 3D scaffold-based models using biomaterials such as polycaprolactone (PCL) and hydrogels. For instance, Yadav et al. [10] utilized macroporous 3D PCL scaffolds to model ionizing radiation-induced senescence in cancer cells, whereas De et al. [11] established a novel 3D PCL-based ex vivo platform to study circulating tumor cells derived from breast cancer patients.

The convergence of 3D printing and biology has given rise to the field of biofabrication, in which living cells are integrated with biocompatible matrices to form 3D tissue-like constructs. Biofabrication combines the core components of tissue engineering, which include cells, scaffolds, and bioactive factors, into an automated process that yields spatially organized biological constructs [12]. Pioneering studies by Atala and colleagues translated functional bladder tissue engineering from preclinical animal studies to the first successful clinical implantation of autologous, scaffold-seeded bladder constructs in pediatric patients [13,14,15,16]. These efforts established the biological and translational foundation for modern 3D bioprinting, which is a specialized form of AM. This technique employs bioinks, consisting of hydrogels or other printable matrices encapsulating living cells and bioactive factors, which are deposited LbL to generate constructs with predefined geometries and spatiotemporally arranged, multicellular architectures that recapitulate complex native tissue organization.

Bioinks constitute the functional core of the bioprinting process and are formulated from a broad spectrum of natural polymers, including collagen, gelatin, gelatin methacryloyl (GelMA), alginate, fibrin, hyaluronic acid, chitosan, and silk fibroin, as well as synthetic polymers such as polyethylene glycol (PEG), polycaprolactone (PCL), and polyvinyl alcohol (PVA), and decellularized ECM (dECM)-derived materials. These materials are engineered as single-component or composite bioinks to balance printability, structural fidelity, cytocompatibility, and biodegradation, and are loaded with diverse mammalian cell types, including primary cells, adult stem cells, and human induced pluripotent stem cells (hiPSCs), to generate cellularized constructs with tissue-appropriate phenotype and functionality [17,18].

3D bioprinting provides unprecedented precision and reproducibility through spatiotemporal control of cell placement, bioink composition, and tissue microarchitecture within tissue constructs [19]. However, the technique encounters inherent challenges in printing very soft biomaterials, such as low-viscosity hydrogels, elastomers, and protein–polysaccharide composites with elastic moduli below 100 kPa [20]. These materials require precise control of gelation kinetics and structural support strategies during fabrication. They also require strict constraints on printing temperature, crosslinking chemistry, and mechanical stress exposure to maintain cell viability and construct integrity [21]. 3D bioprinting is broadly categorized into scaffold-based and scaffold-free modalities. Scaffold-based bioprinting involves the co-deposition of living cells and exogenous biomaterials, known as bioinks (such as hydrogels, collagen, or synthetic polymers), that act as a temporary ECM to provide immediate mechanical support and structural integrity. Conversely, scaffold-free bioprinting obviates the need for exogenous biomaterial carriers altogether by utilizing exclusively cellular building blocks as bioinks, such as multicellular spheroids, tissue fragments, or organoids, which fuse through biological self-assembly driven by cellular adhesion molecules.

A literature search was conducted in PubMed, Scopus, and Web of Science for peer-reviewed articles published mainly between 2015 and 2026, using combinations of the terms 3D bioprinting, bioinks, tissue engineering, regenerative medicine, extrusion bioprinting, droplet-based/inkjet bioprinting, laser-assisted bioprinting, and scaffold-free bioprinting, combined with individual tissue systems such as bone, cartilage, skin, cardiac, vascular, ocular, neural, and dental. Original research articles, peer-reviewed reviews, book chapters, and Research Institutes online news articles published in the English language were included. Priority was given to studies published between 2020 and 2026 to ensure currency, supplemented by earlier landmark studies where historically relevant.

Several comprehensive reviews on 3D bioprinting have appeared recently, typically organized around a single bioprinting modality, a specific surgical speciality, or a system-standardization perspective [22,23,24]. The present review provides a comprehensive correlation between the physicochemical constraints of the principal bioprinting modalities and the tissue-specific bioink and cell-source strategies established for each platform, critically evaluating primary literature through early 2026. This review discusses the classification and operating principles of 3D bioprinting technologies, together with recent advances in their scaffold-based and scaffold-free applications to tissue engineering and regenerative medicine across bone, musculoskeletal, vascular, skin, and other tissue systems.

## 2. Classification of 3D Bioprinting Technologies and Bioinks

3D printing (or additive manufacturing) refers broadly to any layer-by-layer (LbL) fabrication processes, irrespective of material or biological content. Bioprinting is a specialized subset of 3D printing in which bioinks, hydrogels, or other biomaterial matrices containing living cells and/or bioactive factors are deposited to generate biologically functional constructs. Biofabrication is the comprehensive field that incorporates bioprinting alongside bioassembly (the automated, robotic positioning of pre-formed cellular units like spheroids or organoids), both of which require subsequent biological maturation to form functional constructs. Under this biofabrication paradigm, acellular scaffolds are structural constructs fabricated without embedded cells, intended for post-fabrication cell seeding or in vivo cellular infiltration, whereas cell-laden bioinks are formulations in which cells are directly incorporated into the printable matrix prior to or during deposition.

3D bioprinting facilitates the spatially controlled deposition of biomaterials and living cells into organized biological constructs. These technologies are broadly classified into scaffold-based and scaffold-free approaches. Scaffold-based 3D bioprinting includes extrusion-based, droplet-based (including inkjet and drop-on-demand systems), laser-assisted, and light-mediated (photopolymerization-based) bioprinting [25]. Although most of these approaches are based on sequential layer-by-layer (LbL) fabrication, several emerging strategies have substantially expanded the capabilities of the field. Volumetric bioprinting, a tomography-inspired method in which dynamic visible-light projections are applied to a rotating, cell-laden photoresponsive hydrogel reservoir, solidifies the entire construct volume within seconds rather than LbL, enabling rapid generation of centimeter-scale tissue constructs while maintaining high cell viability [26]. More recently, support-bath-assisted (embedded) bioprinting, in which bioinks are extruded directly into a granular hydrogel or microgel suspension medium that provides transient structural support, has emerged as an important complement to conventional LbL deposition, enabling freeform fabrication of low-viscosity or mechanically weak constructs with complex overhanging geometries [27]. Another strategy of 3D bioprinting, which overcomes the necessity for artificial matrices of biomaterial, is scaffold-free bioprinting, a bottom-up tissue assembly approach. This method utilizes living building blocks, predominantly high-density spheroid-based bioinks, which possess fluid-like properties that allow them to fuse naturally through intrinsic cell-to-cell signaling and host-driven ECM secretion. Precise spatial maniulation of these cellular blocks is achieved through advanced robotic systems, including mechanical microneedle arrays via the Kenzan method, vacuum-driven pickup using aspiration-assisted bioprinting (AAB), and contactless cellular manipulation through magnetic bioprinting and other field-directed tissue assembly approaches. Furthermore, this category also encompasses next-generation organoid-based bioprinting, in which structurally complex, stem cell-derived micro-organs, known as Organ Building Blocks (OBBs), are positioned at precise coordinates and subsequently assembled into larger macrotissues through guided fusion [28]. Together, these innovative approaches enable the rapid generation of highly cellularized, anatomically accurate, and structurally complex tissue constructs, thereby advancing the development of functional engineered tissues [26,29,30].

The term “bioink” has evolved beyond any single bioprinting modality and is now generally defined as “a formulation of cells suitable for processing by an automated biofabrication technology that may also contain biologically active components and biomaterials” [31]. In accordance with this paradigm, bioinks can be divided into two broad categories, those consisting of biomaterials containing cells, and those consisting only of cells [32,33].

An ideal bioink for 3D bioprinting should possess high biocompatibility, suitable rheology, structural fidelity, and controlled post-print stabilization while preserving the biochemical and mechanical properties needed for tissue regeneration [34]. It should support high cell viability, enable uniform cell distribution, and allow the printed construct to maintain its geometry during and after fabrication [17,35]. For material extrusion, the bioink must generally exhibit moderate-to-high viscosity, shear-thinning behavior, yield stress, and rapid viscoelastic recovery so that filaments are deposited continuously without collapsing after bioprinting [36]. For droplet-based bioprinting, the formulation should be low-viscosity and homogeneous, and able to form stable droplets without clogging or satellite formation, with secondary crosslinking often required after deposition to improve shape retention [37]. Laser-assisted bioprinting requires a cell-friendly, homogeneous ink that transfers efficiently under laser energy, tolerates high cell loading, and remains compatible with rapid post-print stabilization [38]. Light-based bioprinting requires a photoreactive, optically suitable bioink that crosslinks rapidly and uniformly under light exposure while minimizing phototoxicity and preserving cell function [35] (Table 1). Across all biofabrication modalities, the optimal bioink formulation requires a multi-parametric optimization that balances rheological printability, cytocompatibility, structural mechanical integrity and degradation kinetics tailored to the target tissue.

## 3. Scaffold-Based 3D Printing in Tissue Engineering and Regenerative Medicine

Three-dimensional (3D) bioprinting employs diverse additive manufacturing processes to assemble biomaterial and living cells into complex tissue-like constructs with precise spatial organization [19,39]. Because human tissues exhibit remarkable heterogeneity in structure and function, the fabrication of biomimetic tissue analogues requires specialized, application-tailored fabrication strategies [40]. Bone tissue demands high mechanical strength and sustained load-bearing capability; neural tissue requires a highly hydrated microenvironment to support cellular viability, axonal guidance, and electrochemical signaling; vascular tissues depend on perfusable, hollow-channel architecture; cartilage must exhibit zonal mechanical integrity and resistance to compressive loading; and hepatic tissues require a densely packed, lobular cellular architecture that supports efficient mass transport, nutrient exchange, and metabolic activity [41,42,43,44]. Owing to these diverse and often divergent biological, mechanical, and architectural requirements, the recapitulation of native tissue architecture requires multiple bioprinting modalities capable of utilizing distinct cell populations, bioink compositions, scaffold geometries, porosity gradients, and material properties.

**Figure 1:**
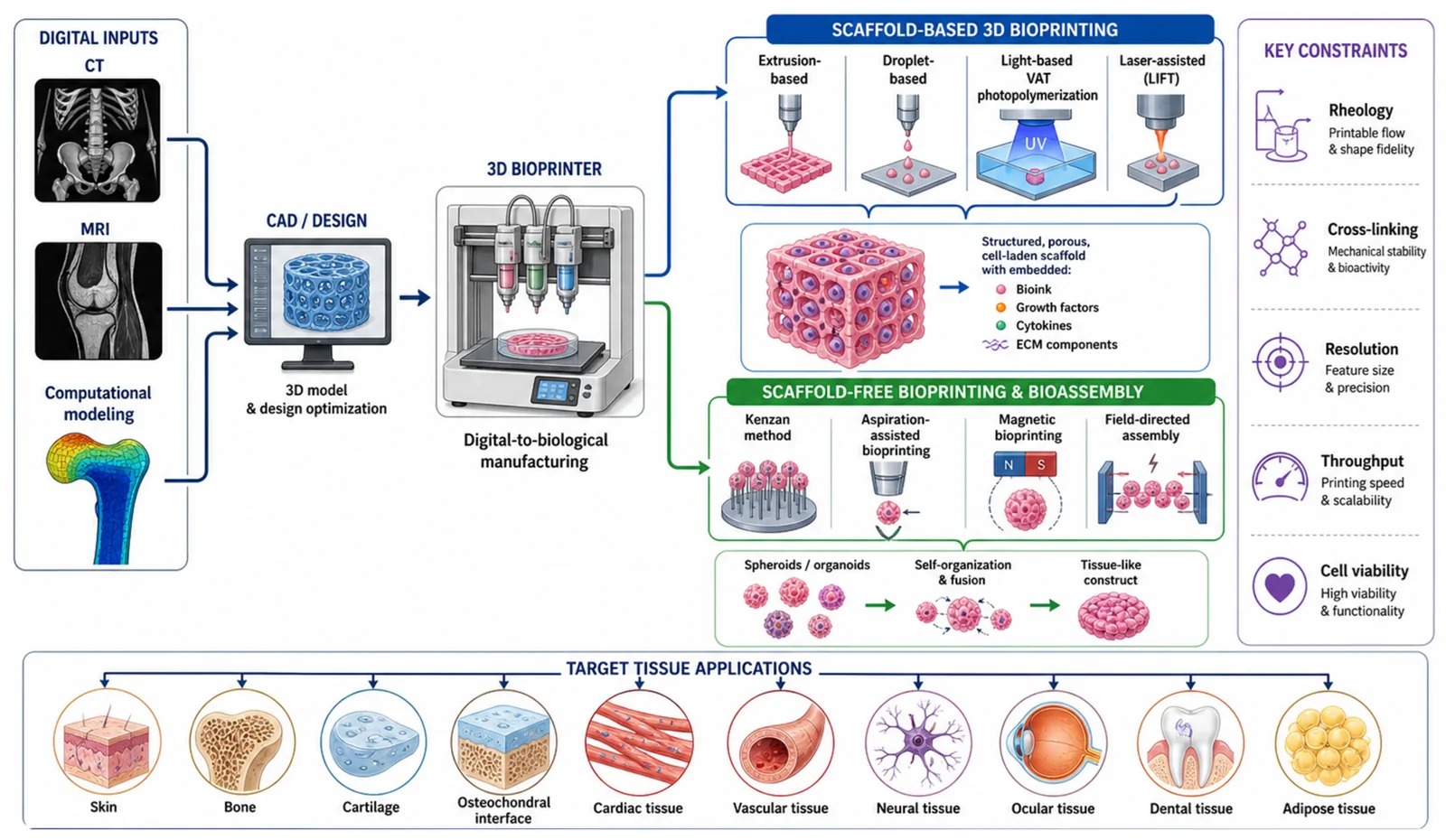
Schematic illustration of 3D bioprinting utilizing scaffold-based (extrusion, droplet, vat photopolymerization, LIFT) and scaffold-free (spheroid/aggregate bioassembly) approaches for diverse tissue engineering applications.

3D printing and bioprinting have evolved into key enabling technologies for fabricating patient-specific scaffolds and cell-laden constructs that better recapitulate native tissue architecture than conventional scaffold fabrication methods, such as solvent casting, freeze-drying, or gas foaming, which offer limited control over internal pore architecture and lack the spatial resolution needed to precisely position cells within the construct [45]. Depending on the underlying printing mechanism, scaffold-based 3D bioprinting techniques are commonly categorized into extrusion-based, inkjet/drop-on-demand, light-based (stereolithography/digital light processing), laser-assisted bioprinting (LaBP), granular or support-bath-assisted (embedded), and hybrid methods that integrate two or more of these modalities within a single fabrication workflow. Each modality has specific constraints on bioink viscosity, crosslinking mechanism and kinetics, spatial feature resolution, throughput, and post-printing cell viability, which in turn determine the choice of biomaterials, applicable cell types, and target tissue applications (Figure 1).

Although printability, shape fidelity, filament/droplet resolution, and structural integrity immediately after fabrication are necessary, they constitute baseline fabrication requirements rather than definitive benchmarks of physiological relevance [35,46]. Biological and functional validation of a scaffold-based 3D bioprinted construct additionally requires post-print cell viability and sustained proliferation over physiologically relevant timescales (days to weeks); maintenance or induction of tissue-specific phenotype, assessed via lineage marker expression (e.g., osteogenic markers ALP/RUNX2 for bone, chondrogenic SOX9/COL2A1 for cartilage) [47,48]; tissue-specific functional assays, such as mineralized matrix deposition for bone, contractility and intracellular calcium transients in cardiac constructs, or barrier/permeability assays for skin and vascular constructs; mechanical properties of the construct that approximate those of the native tissue microenvironment; and host integration and functinal performance in vivo. A 3D bioprinted scaffold-based construct that satisfies printability requirements yet exhibits deficits in these downstream biological functions should be regarded as morphologically biomimetic but physiologically non-functional. Here, we discuss scaffold-based bioprinting modalities and their regenerative tissue engineering applications, in which the bioink itself constitutes the structural scaffold.

### 3.1. Material Extrusion-Based Bioprinting (MEBB)

Material extrusion-based bioprinting (MEBB) is the most extensively utilized bioprinting strategy for fabricating 3D biological constructs, owing to its operational simplicity, broad material compatibility, and capacity to process high cell densities. In this technique, bioinks comprising cells, biomaterials, and bioactive molecules are continuously extruded through a nozzle and deposited according to a computer-aided design (CAD) model, enabling LbL assembly of complex tissue architectures [49,50]. MEBB processes a wide range of bioink viscosities and extrusion pressures, facilitating the fabrication of mechanically stable constructs using hydrogel formulations such as alginate, gelatin, collagen, hyaluronic acid, gelatin methacryloyl (GelMA), and decellularized ECM (dECM)-derived bioinks. MEBB is classified into three major categories, according to the extrusion mechanism, namely pneumatic, piston-driven, and screw-assisted extrusion [51]. Pneumatic extrusion systems utilize compressed air to generate pressure within a cartridge, forcing the bioink through the nozzle; these systems provide rapid pressure control and are particularly suited to shear-thinning bioinks whose viscosity decreases under applied stress. Piston-driven systems employ a mechanical plunger to directly compress the bioink reservoir, providing superior volumetric precision and reduced compressibility artifacts compared with pneumatic systems, making them advantageous for highly viscous formulations. Screw-assisted systems employ a rotating auger mechanism to transport and dispense bioinks and are most commonly applied to composite materials such as ceramic-containing formulations intended for bone implants; however, the mechanical shear generated by the rotating screw can adversely affect the viability of encapsulated cells [52]. Common MEBB implementations, including direct ink writing (DIW), liquid deposition modeling (LDM), freeform reversible embedding of suspended hydrogels (FRESH), coaxial extrusion, and embedded extrusion bioprinting, predominantly employ pneumatic or piston-driven mechanisms, whereas fused deposition modeling (FDM) and fused filament fabrication (FFF) typically utilize screw-assisted or thermoplastic extrusion.

In recent years, conventional extrusion bioprinting has been substantially extended through the development of embedded extrusion, microfluidic-assisted extrusion, coaxial extrusion, and FRESH strategies. These approaches enable the fabrication of vascularized networks, hollow tubular structures, and highly complex freeform geometries that are difficult to achieve with standard LbL deposition. In particular, embedded extrusion employs temporary support matrices that enhance structural fidelity during printing, while coaxial extrusion facilitates the direct fabrication of perfusable channels for vascular tissue engineering [53]. Despite ongoing challenges related to print resolution and shear-induced cellular stress, MEBB remains the most versatile platform for the production of patient-specific engineered tissues and organ substitutes [54,55] (Table 2, Table 3 and Table 4).

#### 3.1.1. Bone Tissue Engineering

Extrusion-based bioprinting has been extensively investigated for the fabrication of silk fibroin (SF)-composite scaffolds in bone regeneration (Table 2). Sangkert et al. [56] produced 3D-printed alginate/poly(vinyl alcohol) (PVA) scaffolds incorporating 2% silk fibroin (SF) to recapitulate the compositional features of bone extracellular matrix (ECM). These scaffolds supported MC3T3-E1 osteoblast adhesion, viability, and proliferation, and demonstrated enhanced alkaline phosphatase (ALP) activity, protein synthesis, and calcium deposition, indicating suitability for maxillofacial bone reconstruction. Earlier investigations established the bone regeneration potential of nanocellulose-based biopolymer scaffolds [112], and Zhang et al. [113] reported the fabrication of aligned cellulose scaffolds loaded with osteogenic growth factors. Patel et al. [57] employed extrusion 3D bioprinting to produce chitosan/silk fibroin/cellulose nanoparticle (CS/SF/CNP) composite scaffolds and characterized their osteo-immunomodulatory responses in RAW 264.7 macrophages. These CS/SF/CNP scaffolds promoted macrophage polarization toward an osteogenic-permissive phenotype and upregulated osteogenesis-associated genes through the mitogen-associated protein kinase (MAPK) pathway, demonstrating superior osteoinductive potential.

To design biomimetic aerogel-based composite scaffolds, Ng et al. [58] synthesized photocrosslinkable methacrylated silk fibroin (SF-MA) and methacrylated hollow mesoporous silica microcapsules (HMSC-MA), incorporating drug-loaded HMSC-MA into self-assembled SF-MA to form a printable composite ink. Scaffolds fabricated via micro-extrusion-based 3D printing of the SF-MA-HMSC system exhibited controlled shape fidelity, sustained antibiotic (ciprofloxacin) delivery, and promoted cellular ingrowth, proliferation, and osteoblastic differentiation, as reflected by upregulation of osteogenic markers and matrix mineralization, thereby supporting their application in bone infection and bone defect therapy.

Additive manufacturing (AM) enables the free-form fabrication of 3D structures with precisely defined external geometries tailored to patient-specific defects and internal pore architectures optimized for bone regeneration. Although collagen is the most abundant ECM protein in bone, its inherently low viscosity and limited printability pose significant challenges for AM-based scaffold fabrication. Lode et al. [59] addressed this by developing a highly viscous, high-density collagen dispersion composed of insoluble collagen fibrils and fibers in their swollen state (pH 4.0) for 3D scaffold fabrication via extrusion-based 3D plotting. The swollen fibrillar state facilitated homogeneous extrusion and the deposition of uniform strands, yielding well-defined 3D scaffolds that were subsequently stabilized by freeze-drying and covalent crosslinking with carbodiimide (EDC). The resulting scaffolds exhibited high shape fidelity, dimensional stability, and hierarchical porosity characterized by interconnected micropores. Human mesenchymal stromal cells (hMSCs) cultured on these scaffolds demonstrated excellent cytocompatibility and retained adipogenic and osteogenic differentiation capacity, demonstrating their potential for adipose and bone tissue engineering.

To overcome the limited mechanical strength of pure collagen scaffolds, which restricts their suitability for complex 3D porous architectures under physiological loading, composite formulations incorporating silk fibroin (SF) have been developed to provide structural reinforcement while preserving biological functionality. Lee et al. [60] utilized a low-temperature extrusion-based 3D printing approach to fabricate porous scaffolds composed of collagen, dECM (to promote cellular activity), and SF (to impart mechanical strength) and demonstrated their capacity to support MC3T3-E1 preosteoblast proliferation and osteogenic differentiation. Building on this platform, low-temperature 3D printing of collagen/dECM/SF (CES) ternary biocomposite scaffolds has been further developed to enhance structural fidelity, mechanical stability, and osteogenic performance of MC3T3-E1 cells, providing an advanced ECM-derived bioink framework for bone tissue engineering [60]. Similarly, Liu et al. [61] demonstrated that low-temperature 3D printing of silk fibroin/collagen/hydroxyapatite (SF/COL/HA) composites yields scaffolds with excellent structural stability and biocompatibility. The mechanical performance and cell-biomaterial interactions of these scaffolds were found to be strongly influenced by pore size, porosity, water absorption capacity, and elastic modulus. Furthermore, the fiber cross-angle structure (FCAS) significantly affected compressive mechanical properties as well as cell adhesion, proliferation, and differentiation potential. A six-layered scaffold configuration with a 90° FCAS markedly enhanced the compressive modulus and promoted MC3T3-E1 proliferation and osteogenic differentiation.

Three-dimensional-printed scaffolds are also employed for controlled drug delivery, an important adjunct to scaffold-mediated tissue regeneration. Adrenergic β-receptor blockade in traumatic bone defects has been shown to promote bone regeneration, and Wu et al. [62] investigated the relative efficacy of systemic versus local, sustained release of the β-blocker propranolol delivered through filament-free 3D-printed collagen/PVA/propranolol/hydroxyapatite (CPPH) composite scaffolds implanted into distal femur defects in rats. Compared with intraperitoneal propranolol injection, scaffold-based local delivery enhanced new bone formation, osteogenic differentiation, and migration of bone marrow stromal cells (BMSCs), while inhibiting osteoclastogenesis of bone marrow monocytes (BMMs) through suppression of β-receptor activation, as confirmed by micro-CT analysis and histological examination.

In cell-printing applications, bioink composition critically determines the fabrication quality of microscale and macroscale cell-laden constructs. Alginate-based bioinks are widely used due to their biocompatibility, low cytotoxicity, printability, and ease of ionic crosslinking with divalent cations; however, they lack intrinsic cell-adhesive motifs and biological signaling cues. To address this, Yeo and Kim [63] developed collagen-based bioinks incorporating human adipose-derived stem cells (hASCs) crosslinked with the non-toxic plant polyphenol tannic acid (TA). Macroscale 3D porous cell-laden collagen/TA constructs demonstrated significantly higher metabolic activity, including cell viability and proliferation, relative to alginate controls, suggesting that collagen/TA bioinks are promising ECM-mimetic alternatives for 3D tissue regeneration. Extending this approach, Lee et al. [114] applied TA crosslinking (0.1–3 wt%) to high-density collagen bioinks (5 wt%) encapsulating MC3T3-E1 preosteoblasts, producing 3D mesh constructs with well-defined pore architectures, substantially enhanced mechanical properties, and sustained high cell viability (~95%) in vitro.

Kim et al. [64] fabricated a highly porous, biocompatible, and cell-laden 3D macroscopic construct (21 × 21 × 12 mm^3^) using a collagen bioink (5 wt%, pH 7.1) crosslinked with genipin (~1 mM, 1 h) for hard tissue regeneration. The pore-interconnected constructs (pore sizes > 400 µm) encapsulating osteoblast-like MG63 cells and human adipose-derived stem cells (hASCs) maintained high cell viability and exhibited significant osteogenic differentiation, evidenced by elevated ALP activity, calcium deposition, and upregulated expression of bone morphogenetic protein-1 (BMP-2), Runt-related transcription factor 2 (RUNX2), type I collagen (Col-I), and osteocalcin (OCN), with Col-I and OCN expression reaching 1.39-fold and 1.64-fold, respectively, relative to alginate mesh controls.

More broadly, the implementation of 3D-printed scaffolds has emerged as a transformative strategy to regulate gene expression across multiple phases of bone repair [115,116]. During osteogenesis, mesenchymal stem cells (MSCs) sequentially differentiate into preosteoblasts and mature osteoblasts that synthesize the osteoid matrix, which subsequently undergoes biomineralization. The expression of osteogenic markers, including ALP, RUNX2, type I collagen alpha I chain (COL1A1), osteopontin, and osteocalcin, is profoundly influenced by the physicochemical and biochemical properties of the scaffold and its local microenvironment. ALP is upregulated during early preosteoblastic activity, whereas COL1A1 expression increases during osteoprogenitor maturation toward a committed osteoblastic phenotype [117,118]. The initial stages of bone regeneration additionally involve transcriptional upregulation of genes governing cell adhesion, migration, immunomodulation, and angiogenesis, which are coordinated with programs controlling cell fate determination, matrix deposition, and osteogenic maturation [119].

**Figure 2:**
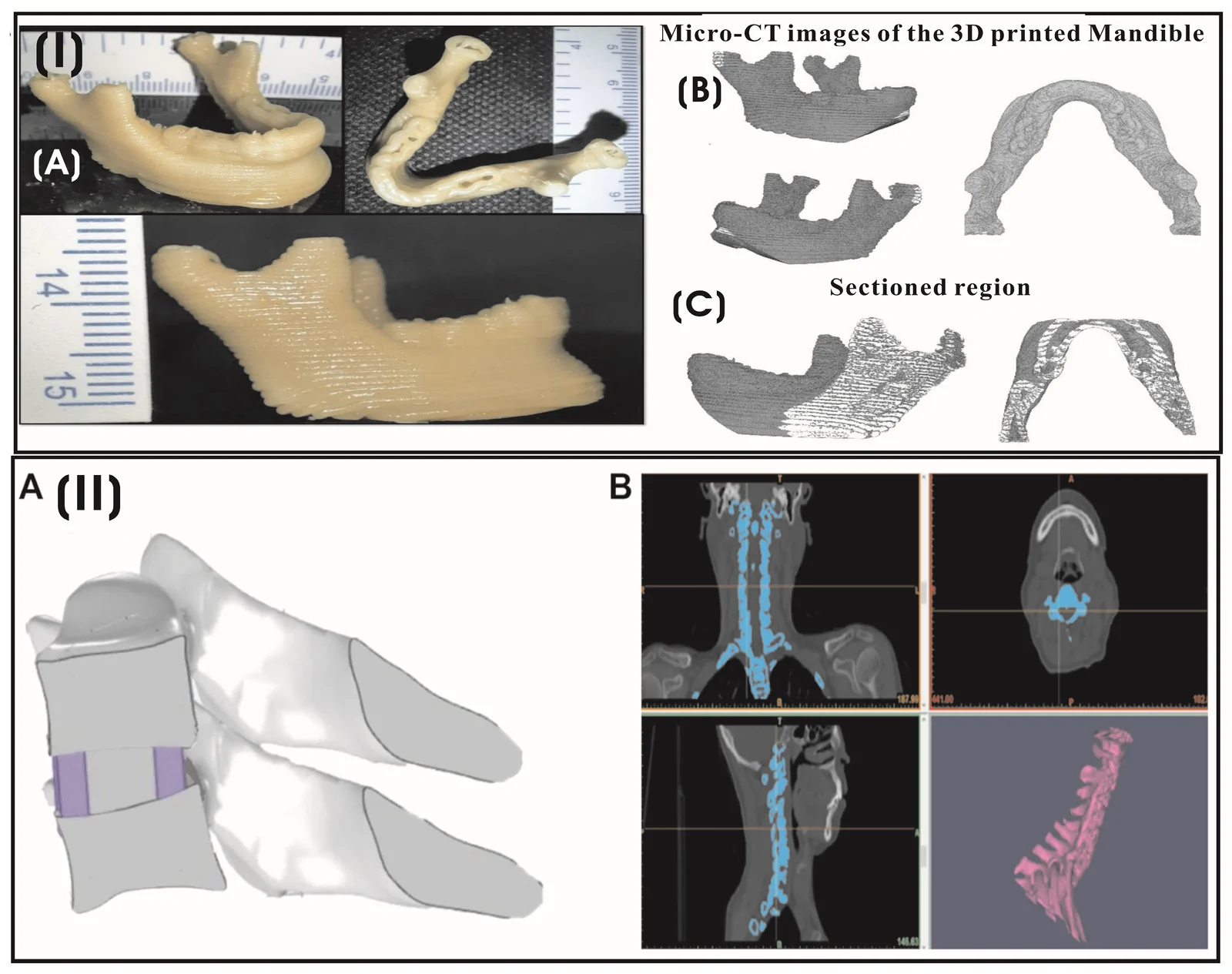
(**I**) A 3D model generated based on patient CT data was utilized to 3D print a patient defect-specific model (mandible) with PCL–40AM composite: (**A**) digital photographs; (**B**,**C**) micro-CT images. Reprinted with permission from [65]. Copyright 2022 American Chemical Society. (**II**) (**A**) 3D model of the cervical interbody fusion cage obtained by Mimics. (**B**) Original 3D CT image of the cervical spine scanned from a healthy woman. Reproduced from [85] under the terms of the Creative Commons Attribution License (CC BY).

Among natural biomaterials, silk proteins (SPs) derived from *Bombyx mori* are recognized for their superior mechanical strength, cytocompatibility, and immunomodulatory properties, establishing them as attractive components for next-generation 3D-printed scaffolds for bone tissue engineering. Polycaprolactone (PCL) scaffolds reinforced with silk fibroin microfibers significantly enhanced adhesion and osteogenic differentiation of human gingival mesenchymal stem cells (hGMSCs) [65] (Figure 2(I)). In a rabbit calvarial defect model, PCL–SF composite scaffolds promoted early cell adhesion and proliferation, achieving 47.4–50.3% bone growth at six weeks, approximately twice that of PCL alone (16.7–19.9%), and 80–87.3% new bone formation at twelve weeks, representing a fourfold increase over the PCL control group (18.6–22.4%) [66]. Similarly, 3D porous silk scaffolds incorporating the histone deacetylase 2/3 (HDAC2/3) inhibitor MI192 promoted bone-like tissue formation by human dental pulp stromal cells (hDPSCs), demonstrating that epigenetic reprogramming within 3D silk scaffolds further enhances osteogenic efficacy [67]. Vyas et al. [68] prepared *Bombyx mori* silk microparticles (SMPs) by milling silk fibers and incorporating them as reinforcing agents in PCL/SMP composites. At 10 wt% SMP loading, scaffolds demonstrated improved compressive Young’s modulus, mechanical stability, and hydrophobicity after 21 days of culture, with elevated metabolic activity, cell viability, migration, and calcium deposition by human adipose-derived mesenchymal stem cells (hADMSCs), confirming osteogenic potential.

**Figure 3:**
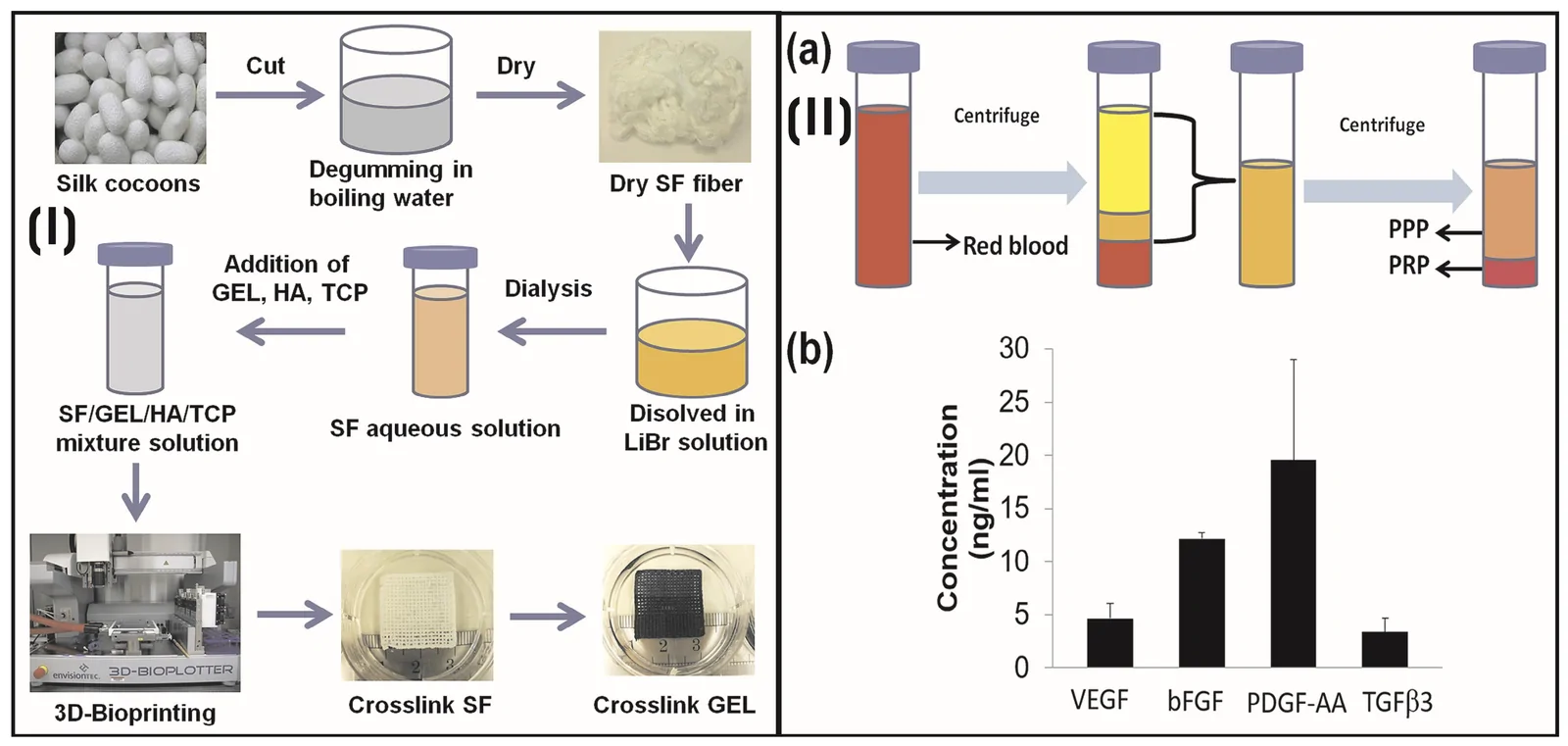
(**I**) Schematic illustration of the fabrication of the SF-based hybrid scaffold. (**II**) (**a**) Schematic diagram of the preparation process of PRP, (**b**) the concentration of different growth factors in PRP (*n* = 3). Reprinted from [69], Copyright © [2019] The Authors. Published by Elsevier B.V. on behalf of KeAi Communications Co., Ltd. under a Creative Commons CC BY License.

To achieve patient-specific architectures with enhanced biological functionality, Wei et al. [69] formulated a composite bioink consisting of SF, gelatin (GEL), hyaluronic acid (HA), and tricalcium phosphate (TCP), crosslinked with ethanol and genipin, to produce dual-crosslinked SF/GEL/HA/TCP scaffolds. When supplemented with human platelet-rich plasma (PRP), these scaffolds promoted hADMSC proliferation and upregulation of late osteogenic markers, demonstrating improved osteoinductive capacity (Figure 3). Liu et al. [70] developed a novel bioink composed of SF, gelatin, and propanediol (polyol) with optimized rheological and mechanical stability, and demonstrated that 3D bioprinted constructs enhanced osteogenic-specific gene expression in MC3T3-E1 preosteoblasts through Smad1/5/8 and Runx2 signaling pathways. To further mimic the mineral phase of native bone, Huang et al. [71] synthesized SF/hydroxyapatite (SF/HA) nanocomposites via in situ mineral precipitation dispersed in sodium alginate (SA) bioink for 3D printing. The resulting porous SF/HA–SA scaffolds (interconnected pores ~400 µm, ~70% porosity, compressive strength > 6 MPa) supported in vitro apatite formation, degradation, and hBMSC proliferation and differentiation. Additionally, Kim et al. [120] developed silk fibroin-based bioabsorbable fixation systems using centrifugal casting, demonstrating their applicability as 3D-printed internal fixation devices such as plates and screws for bone fracture stabilization.

Scaffold-mediated tissue engineering represents a promising strategy for regenerating critically sized bone defects with limited intrinsic self-healing capacity. An ideal bone tissue engineering scaffold must integrate biological functionality, optimized microarchitecture, and physiologically relevant mechanical properties to support cellular infiltration, vascularization, and de novo tissue formation. Consequently, Karamat-Ullah et al. [72] developed an antibacterial, biocompatible silica-silk fibroin (SiO_2_-SF) gel-based bioink via a sol–gel synthesis coupled with SF self-assembly, followed by micro-extrusion-based 3D printing and freeze-drying to produce hierarchically porous hybrid aerogel scaffolds. Cell adhesion and antibacterial activity were significantly enhanced by covalent conjugation of thiol-terminated antimicrobial/cell-adhesive peptide sequences (SH-CM-RGD) to the SiO_2_-SF matrix, accomplished via pre-print modification (prior to sol–gel processing) or post-print functionalization (directly on 3D-printed SiO_2_-SF gels) strategies. The resulting 3D-printed SiO_2_-SF hybrid aerogel constructs exhibited superior mechanical properties, broad-spectrum bactericidal activity against both Gram-positive and Gram-negative bacteria, and enhanced osteoconductivity, demonstrating potential for bone defect repair and infection-resistant orthopedic applications.

**Figure 4:**
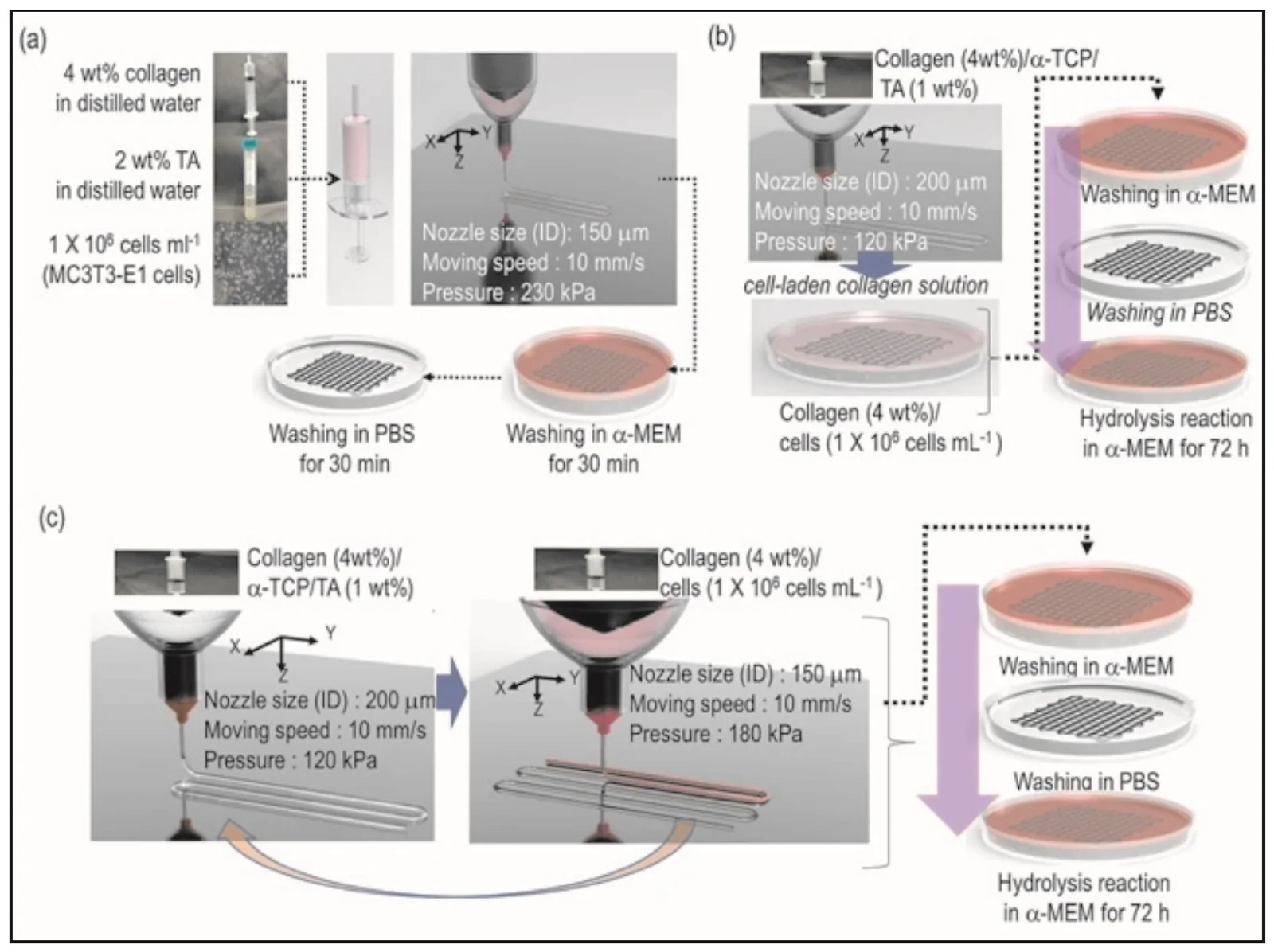
Fabrication schematics of cell-loaded scaffolds: (**a**) A cell-laden collagen scaffold, (**b**) a cell-laden α-TCP/collagen scaffold loaded using a dipping method, and (**c**) a 3D cell-laden α-TCP/collagen scaffold loaded using cell printing. Reproduced from [74], licensed under CC BY 4.0.

For the successful regeneration of tissues, biomedical scaffolds must exhibit adequate mechanical properties to maintain structural integrity under complex biophysical and biochemical conditions. In addition, they should possess a highly porous architecture, excellent biocompatibility, and controlled biodegradability without generating cytotoxic degradation by-products [121]. Incorporating synthetic polymers, such as poly(ε-caprolactone) (PCL) and poly(lactic acid) (PLA), together with bioceramics like hydroxyapatite (HA) and tricalcium phosphate (TCP), enhanced both the mechanical performance and osteoinductive properties of bioprinted scaffolds [122,123]. Raja and Yun [73] demonstrated a 3D-printed core/shell cell-laden construct in which an α-TCP ceramic core was fabricated using a screw-rotating extrusion system, while the shell comprised alginate hydrogel encapsulating MC3T3-E1 cells deposited via pneumatic dispensing. This dual-component design enhanced mechanical robustness and supported sustained cell viability during long-term in vitro culture [73]. Kim et al. [74] subsequently developed multilayered 3D bioceramic cell-laden scaffolds composed of α-TCP/collagen with ceramic volume fractions exceeding 70%, fabricated via a two-step, three-axis robot extrusion-based 3D printing process, yielding constructs with improved mechanical strength and elevated metabolic activity and mineralization of preosteoblasts (MC3T3-E1), facilitating bone tissue regeneration (Figure 4). In another study, Neufurth et al. [75] demonstrated that 3D-bioprinted alginate/gelatin matrices laden with SaOS-2 osteoblast-like cells, overlaid with agarose and polyphosphate calcium complexes (polyP.Ca^2+^-complex), significantly enhanced cell proliferation and mineralization, further supporting the utility of composite bioceramic bioinks in bone tissue engineering.

Low-temperature additive manufacturing (LT-AM) offers an effective strategy for incorporating thermolabile bioactive molecules and drugs into 3D scaffolds that closely recapitulate native bone tissue. Using collagen/hydroxyapatite (CHA) composite biomaterial ink printed under low-temperature conditions via a freeform fabrication (FFP) printer, Lin et al. [76] fabricated 3D CHA scaffolds with interconnected pore architectures (~600 µm rod diameter) that supported BMSC proliferation, enhanced osteogenic differentiation in vitro, and promoted superior bone repair and graft-to-bone integration in a rabbit femoral condyle defect model relative to non-printed CHA controls. Extending the LT-AM approach, Li et al. [77] prepared type I collagen (CoL)-based bioinks containing either nanohydroxyapatite (nHA/CoL) or deproteinized bovine bone (DBB/CoL), demonstrating that both formulations supported proliferation and osteogenic differentiation of human bone marrow-derived MSCs (hBMSCs) and are viable candidates for 3D-printable osteoconductive bioinks.

Bioactive ceramics such as hydroxyapatite (HAp), tricalcium phosphate (TCP), and bioactive glasses are widely incorporated into 3D-printed constructs to mimic the mineral composition of bone and provide osteoconductive cues. These materials are typically processed as particulate fillers in hydrogel-based bioinks or as self-hardening pastes for direct ink writing (DIW) and fused filament fabrication (FFF), enabling fabrication of porous scaffolds with tailored mechanical properties and interconnected architectures suited to bone regeneration [124,125]. Direct ink writing (DIW), also known as robocasting, enables the extrusion of highly loaded ceramic or glass inks into 3D lattices with controlled porosity and structural geometry. Accordingly, Midha et al. [79] developed hybrid SF-gelatin-bioactive glass (SF-G-BG) inks for DIW, combining the toughness and processability of natural polymers with the osteogenic ion-release capacity of bioactive glass. Calcium ions released under physiological conditions serve as essential mediators of apatite nucleation and mineralized tissue formation. The ions released, particularly calcium and strontium from strontium-substituted glass compositions, induced conformational transitions in Bombyx mori SF from random coil to β-sheet, influencing ink rheology, mechanical stability, and print fidelity. Three-dimensional-printed SF-G-BG constructs cultured with immortalized human bone marrow-derived MSCs (TVA-BMSCs) demonstrated that strontium-incorporated formulations significantly enhanced osteogenic differentiation over 21 days, evidenced by elevated transcription of osteoblast markers (RUNX2, ALP, OPN, ON, IBSP, OCN), osteocyte-related genes (PDRN, DMP1, SOST), and activation of BMP-2, BMP-4, and Indian hedgehog (IHH) signaling pathways. These results demonstrate that strontium-incorporated bioactive glass within DIW-printed SF-G-BG constructs provides a biochemically active and mechanically stable microenvironment that directs stem cell commitment toward osteogenesis, demonstrating the potential of DIW-printable silk-bioactive glass composites as next-generation bone tissue engineering scaffolds. Du et al. [80] developed mesoporous bioactive glass/silk fibroin (MBG/SF) composite scaffolds using integrated printing methodologies; subcutaneous transplantation of hBMSC-laden MBG/SF scaffolds in nude mice revealed marked upregulation of key osteogenic genes (COL-1, BSP, BMP-2, OCN), confirming enhanced osteogenicity in an in vivo environment. These findings demonstrated that appropriately formulated glass and calcium phosphate inks can yield highly porous, mechanically competent scaffolds that support bone formation in load-bearing sites. Recent advancements in DIW have further advanced scaffold design by incorporating triply periodic minimal surface (TPMS) architectures with graphene oxide (GO)-reinforced bioactive glass composites to significantly enhance the mechanical robustness and biological performance of 3D-printed scaffolds for biomedical applications [81].

Copper-doped bioactive glass (CU-BG) has been used to integrate angiogenic and osteogenic functions into DIW-printed constructs. Copper, an essential micronutrient, promotes tissue regeneration by supporting cell migration, angiogenesis via copper transporters and chaperones, and osteogenic differentiation of BMSCs [126,127,128,129]. Dai et al. [82] prepared monodispersed micro-nano Cu-BG particles with a mesoporous structure and varying copper content via a sol–gel and templating approach. In vitro studies showed that Cu-BG enhanced angiogenesis by inducing a pro-inflammatory environment and activating HIF-1α signaling in human umbilical vein endothelial cells (hUVECs), while 2Cu-BG formulations promoted osteogenic differentiation of mouse BMSCs. Tyramine-modified gelatin/silk fibroin/Cu-BG (Gel/SF/Cu-BG) inks were then DIW-printed into 3D scaffolds for implantation into rat bone defects, where Cu-BG-containing constructs promoted angiogenesis and osteogenesis through HIF-1α and TNF-α signaling pathways, supporting their use in a broad range of bone defect repair applications.

Hydrogel composite bioinks containing bioceramics such as hydroxyapatite (HA), α- and β-tricalcium phosphate (TCP), or bioactive glass effectively combine the bioactive mineral phase responsible for osteoconductivity with the hydrated, cell-compatible microenvironment of hydrogel matrices. Sadat-Shojai et al. [78] successfully incorporated nanohydroxyapatite (nHA) into a photocrosslinkable gelatin matrix to develop 3D protein-based hydrogels that exhibited homogeneous mineralization throughout the network after incubation in simulated body fluid (SBF), with enhanced scaffold stiffness and osteoconductivity promoting bone formation in vivo. Despite these advantages, the inclusion of ceramic particles often increases bioink viscosity and abrasiveness, potentially inducing excessive shear stress on cells during extrusion and compromising the structural integrity of the printed constructs. To mitigate these effects, Raja and Yun [73] combined 3D printing, cell printing, and a self-setting calcium phosphate reaction to fabricate core/shell-structured scaffolds consisting of a calcium-deficient hydroxyapatite (CDHA) core and an alginate hydrogel shell laden with MC3T3-E1 pre-osteoblasts, achieving >90% cell viability over a 3-day culture period. Kim and Kim [83] further designed fibrillated collagen/β-TCP composite hydrogel bioinks capable of forming porous cell-laden 3D constructs with excellent mechanical stability, in which encapsulated human adipose-derived stem cells (hASCs) demonstrated robust proliferation and spontaneous osteogenic differentiation without exogenous osteoinductive supplements, indicating that β-TCP effectively triggered osteogenesis.

To address the low viscosity of collagen hydrogel matrices and improve printability, Kajave et al. [84] formulated a bioactive composite ink comprising Bioglass 45S5 (BG) dispersed in methacrylated collagen (CMA). Uniform BG distribution improved the rheological and mechanical properties of the composite, facilitating precise 3D printing and yielding BG-CMA constructs with accelerated mineral deposition, enhanced bone bioactivity in SBF, high hMSC viability, and upregulated ALP activity, validating their osteoinductive potential. 3D printing has become a transformative platform for fabricating orthopedic implants and fixation devices, offering precision design, mechanical tunability, and patient-specific customization. Among various additive manufacturing methods, fused filament fabrication (FFF), a form of extrusion-based 3D printing that uses thermoplastic filaments to build 3D structures LbL, allows the incorporation of bioactive ceramic fillers to create composite materials with tailored mechanical and biological properties. In the context of spinal and fracture repair, bioactive polymer/ceramic constructs have been developed using combinations of biodegradable polymers such as polylactic acid (PLA), polycaprolactone (PCL), and natural proteins such as silk fibroin (SF), integrated with hydroxyapatite (HA) or nano-hydroxyapatite (nHAp) to enhance osteoconductivity and load-bearing capacity.

For spinal fusion applications, cervical interbody fusion cages for anterior cervical discectomy and fusion (ACDF) surgeries, for treating conditions such as cervical spondylosis and herniated discs, have been fabricated from stretchable silk fibroin/nano-hydroxyapatite (SF/nHAp) composites via 3D printing. The resulting constructs exhibited tunable mechanical strength and toughness suitable for physiological loading. Finite element analysis (FEA) demonstrated that SF/nHAp composite cages produce a more uniform stress distribution in vivo than conventional titanium alloy cages, suggesting potential to reduce stress shielding and improve osseointegration [85] (Figure 2(II)). These findings establish SF/nHAp composites as promising candidates for bioresorbable interbody fusion cages and other bone regenerative applications. In fracture fixation, FFF-printed polymer/ceramic composites have demonstrated comparable outcomes. Pitjamit et al. [86] developed hybrid PLA/PCL/HA filaments to fabricate interlocking intramedullary nails for canine diaphyseal fractures, designed to replace bioinert metallic implants. The printed constructs were subsequently coated with silk fibroin via lyophilization to enhance their biological properties. Among the tested formulations, the PLA/PCL/15HA group exhibited the highest compressive strength (82.72 ± 1.76 MPa) and the lowest tensile strength (52.05 ± 2.44 MPa), demonstrating HA’s capacity to improve mechanical integrity. Additionally, HA significantly promoted bone cell proliferation, an effect further enhanced by the silk fibroin surface coating, confirming the bioactivity of the composite structures. Beyond mechanical reinforcement and drug delivery, hydrogel coatings have also been used to improve the corrosion resistance and osteointegration potential of 3D-printed metallic implants. For example, Bordbar-Khiabani et al. [130] showed that octacalcium-phosphate-laden alginate (Alg/OCP) hydrogel coatings on 3D-printed titanium (Ti) substrates increased hydrophilicity and shifted the corrosion potential toward more noble values under simulated inflammatory conditions. The OCP particles in the Alg hydrogel matrix noticeably increased the interfacial charge transfer resistance at the substrate and coating interface, demonstrating a complementary, implant-protective function for hydrogel coatings alongside their established role in bioactive-ion and drug delivery. Similarly, Yeon et al. [87] fabricated PLA/HA/silk composite bone clips for femoral fracture fixation using an FFF-style approach. These 3D-printed clips displayed high biocompatibility, improved mechanical stability, and precise fracture alignment without the need for bone drilling, enabling minimally invasive, patient-customized implantation. Collectively, FFF-fabricated polymer/ceramic composites, particularly when reinforced or surface-coated with silk fibroin or bioactive glass, constitute a versatile and biofunctional platform for developing resorbable fixation devices, spinal implants, and load-bearing scaffolds. Their combination of tunable mechanical behavior, enhanced bioactivity, and architectural precision makes them highly promising for next-generation orthopedic and reconstructive applications. Taken together, the extrusion-based bone bioink systems summarized in Table 2 illustrate a consistent trade-off between mechanical reinforcement and cytocompatibility, in that silk fibroin- and hydroxyapatite-reinforced composites achieve higher compressive strength than pure collagen- or alginate-based inks but generally report lower initial cell viability immediately post-printing. Notably, few of the cited studies report viability beyond 7–14 days in vitro or validate osteogenic outcomes in a load-bearing in vivo model, limiting direct comparison of long-term functional performance across different bioinks.

#### 3.1.2. Cartilage Tissue Engineering

Granular hydrogels comprising densely packed hydrogel microgels represent an emerging class of 3D extrusion bioinks with intrinsic shear-thinning behavior and rapid self-healing properties [131,132]. However, conventional granular systems are limited by large microgel sizes (>100 µm) and low packing densities, which restrict print resolution and reduce construct mechanical strength. Chu et al. [88] developed whey protein microgel-based granular hydrogels (WMGHs) with size-controllable microgels (1, 6, and 20 µm) via protein-polysaccharide segregative phase separation. Smaller microgels enabled WMGHs to behave as continuous liquid inks under appropriate printing conditions, achieving high-resolution (~200 µm) printing with minimal ink spreading (~5%) using a 260 µm nozzle and enabling fabrication of intricate ear and aortic valve models with high geometric fidelity. Incorporation of polyacrylamide (PAM) as a secondary percolating polymeric network transformed WMGH inks into stretchable, tough double-network WMGHs (DN-WMGHs) with up to 36-fold increased toughness (1.45 MJ/m^3^) relative to PAM hydrogels alone. Using this microgel-in-microgel embedded 3D printing technology, DN-WMGH (Double-Network Water-Microgel Hydrogel) was used to fabricate anatomically intricate nasal lateral cartilage constructs with high structural fidelity. The high-resolution extrusion 3D printing of DN-WMGH, combined with its mechanical tunability through controlled microgel size, enables replication of a broad spectrum of native tissue stiffness, from soft brain tissue (<10 kPa) to elastic intestinal tissue (≈300 kPa), suggesting its potential for patient-specific reconstructive surgery, tissue implantation, and drug delivery applications (Table 3).

The fabrication of permeable 3D biodegradable scaffolds provides an essential structural framework supporting cellular attachment, proliferation, and ECM production, ultimately leading to the formation of tissue with native-like biological architecture [133,134]. Despite these advantages, achieving load-bearing capability and geometric fidelity in engineered cartilage tissues remains a major challenge, as it requires constructs with appropriate mechanical properties and spatial organization. Conventional fabrication techniques often fail to reproduce the spatially heterogeneous structures that mimic the complex composition, cellular density, and arrangement observed in native tissues [135]. Three-dimensional bioprinting offers a powerful platform for the spatially controlled co-deposition of biomaterials and living cells, enabling fabrication of heterogeneous constructs with intricate structural and functional properties. When employing ECM-derived proteins such as collagen, their concentration plays a critical role in determining the shape fidelity and mechanical integrity of the printed constructs. High-density type I collagen gels (10–20 mg/mL) exhibited superior mechanical performance and are therefore suitable for bioprinting applications involving soft tissue regeneration [136,137]. Rhee et al. [89] further demonstrated that 3D-printed soft tissue constructs utilizing high-density collagen hydrogels encapsulating primary meniscal fibrochondrocytes exhibited excellent geometric precision, high cell viability, enhanced mechanical strength, and well-defined microstructural organization.

Hydrogels such as alginate, collagen, and agarose have garnered significant attention as promising bioink matrices owing to their inherent biocompatibility, minimal cytotoxicity, and high water content, which together recapitulate the physicochemical characteristics of the native ECM. Among them, alginate is widely employed in LbL bioprinting due to its rapid ionic crosslinking in the presence of calcium and other divalent cations. Daly et al. [90] evaluated several hydrogel-based bioinks, including agarose, alginate, gelatin methacrylate (GelMA), and commercial BioINK^TM^, for their extrusion printability and capacity to support cartilage tissue formation in vitro. Their findings revealed that alginate and agarose matrices promoted the formation of hyaline cartilage-like tissue rich in type II collagen, whereas GelMA and the polyethylene glycol methacrylate (PEGMA)-based BioINK^TM^ facilitated the formation of fibrocartilage-like tissue containing both type I and type II collagens. GelMA exhibited superior printability and structural fidelity compared to alginate and agarose bioinks. To further enhance the mechanical performance and print resolution, Muller et al. [91] reported sodium alginate-nanocellulose composites as bioinks for extrusion-based 3D bioprinting of cartilage constructs.

Although alginate is widely employed as a bioink in 3D bioprinting, its bio-inertness and lack of cell-adhesive motifs limit its capacity to support cell attachment and proliferation. Consequently, alginate-based composites incorporating bioactive polymers have been developed to impart biochemical functionality that promotes cellular adhesion, proliferation, and differentiation. For instance, composite hydrogels composed of sodium alginate (SA) and collagen (COL) have been reported to enhance proteoglycan synthesis and chondrocyte proliferation [138]. When utilized as bioinks for extrusion-based 3D bioprinting of chondrocytes using a 3D Bioplotter system, SA/COL composites exhibited improved mechanical strength while facilitating cell adhesion and proliferation. Moreover, these hybrid hydrogels promoted the expression of cartilage-specific markers, including aggrecan (*Acan*)*,* collagen type II alpha 1 (*Col2a1*), and sex-determining region Y-box9 (*Sox9*), while downregulating the fibrocartilage marker *Col1a1*, thereby preserving the chondrocytic phenotype and preventing dedifferentiation [92].

For LbL bioprinting of complex, cell-laden hydrogel constructs, the choice of crosslinking strategy must ensure structural stability without cytotoxic effects. Among available strategies, photo-initiated crosslinking is widely utilized owing to its spatiotemporal control. Alternatively, supramolecular host–guest chemistry has recently emerged as a complementary biocompatible crosslinking approach; Park et al. [139] reported a non-covalent crosslinking system based on the highly specific host–guest interaction between cucurbit[n]uril (CB) and polyamine (PA), forming reversible yet stable hydrogels. Building upon this concept, Jung et al. [93] developed a 3D supramolecular hydrogel composed of monofunctionalized CB-conjugated hyaluronic acid (CB-HA) and diaminohexane-modified HA (DAH-HA). These monoCB/DAH-HA hydrogels exhibited tunable crosslinking density, favorable mechanical characteristics, and high-water solubility, effectively supporting chondrogenic differentiation of human mesenchymal stem cells (hMSCs).

The FRESH (freeform reversible embedding of suspended hydrogels) technology has significantly extended the capabilities of extrusion-based bioprinting for fabricating complex, cell-laden constructs with high spatial resolution and improved structural fidelity, particularly for low-modulus materials that are difficult to process by conventional methods. As introduced by Hinton et al. [94] from Feinberg’s group, FRESH employs a support-bath-assisted approach in which hydrogels, including alginate, type I collagen, and fibrin, with elastic moduli below 500 kPa, are printed within a thermoreversible gelatin microparticle bath at approximately 200 µm resolution using computer-aided design (CAD). This approach has been applied to nasal cartilage engineering, where grafts must withstand static gravitational loading and wound contracture as well as dynamic muscular forces. Cell-based tissue-engineered grafts are often preferred over autografts or allografts, as they reduce donor-site morbidity and infection risk. Andrew et al. [95] used a cell-based tissue engineering strategy to reconstruct nasal cartilage by bioprinting human nasal chondrocytes within a type I collagen hydrogel on a clinically approved semi-porous type I/III collagen hybrid membrane scaffold via a CAD-based LbL printing process, achieving high spatial resolution and homogeneous cell distribution.

Because native collagen hydrogels exhibit low viscosity, limited elastic modulus, and slow gelation kinetics, FRESH has been used to overcome these limitations in nasal cartilage bioprinting. Lan et al. [96] premixed monolayer-expanded human nasoseptal chondrocytes (hNCs) with pH-neutral type I collagen and printed constructs within a gelatin support bath maintained at room temperature. Upon transfer to 37 °C, collagen fibrillogenesis was induced while the gelatin bath gradually melted, enabling the formation of stable cell-laden constructs for chondrogenic maturation. In a subsequent study, Lan et al. [97] combined CT-based patient-specific modeling with an hNC-laden type I collagen hydrogel to fabricate customized lower lateral cartilage grafts for nasal reconstructive surgery. Collectively, these studies demonstrate that support-bath-assisted bioprinting can generate anatomically matched, cell-rich cartilage constructs with enhanced feasibility for clinical translation.

Therefore, granular WMGH systems achieve superior print resolution (~200 µm) and tunable mechanical toughness but remain at an early stage of development, whereas alginate and agarose bioinks promote hyaline-like, type II collagen-rich tissue at the expense of printability, a trade-off that GelMA/PEGMA systems largely invert, favoring printability over chondrogenic fidelity. High-density collagen and FRESH-based printing offer the closest anatomical fidelity for clinical grafts, though native-tissue mechanical matching remains a challenge. No single platform provides resolution, mechanical performance, and chondrogenic fidelity simultaneously, demonstrating the need for hybrid, composite bioink strategies.

#### 3.1.3. Musculoskeletal Tissue Engineering

The fabrication of customized 3D scaffolds for osteochondral tissue regeneration requires bioprinting of heterogeneous, multilayered hydrogel constructs that spatially integrate the distinct mechanical and biochemical requirements of both bone and cartilage compartments. Shim et al. [98] demonstrated the use of human turbinate-derived mesenchymal stromal cells (hTMSCs), a multipotent cell source with chondrogenic, osteogenic, and adipogenic differentiation capacity, within multilayered 3D constructs. These constructs utilized a supramolecular host–guest hydrogel composed of mono-cucurbit [6]uril and 1,6-diaminohexane-conjugated hyaluronic acid (monoCB [6]/DAH-HA), along with pepsin-treated collagen (atelocollagen) to facilitate lineage-specific differentiation. When implanted into rabbit knee defects, these constructs successfully promoted osteochondral tissue regeneration, demonstrating the feasibility of this spatially defined bioprinting strategy for the repair of complex, multi-tissue interface defects (Table 3).

#### 3.1.4. Skin Tissue Engineering

Extrusion-based 3D bioprinting is the most widely used technique in skin tissue engineering due to its simplicity, scalability, and ability to deposit highly viscous, cell-laden bioinks at clinically relevant cell densities (Table 4). In a typical setup, bioinks are extruded through nozzles and deposited LbL, achieving feature sizes on the order of 100–300 µm depending on nozzle diameter and process parameters. Bioink printability is primarily governed by viscosity and rheological behavior; excessively high viscosity can induce damaging shear stresses and compromise cell viability, whereas low viscosity fails to maintain 3D shape fidelity. Ideal extrusion bioinks for skin applications therefore exhibit shear-thinning behavior, appropriate yield stress, and rapid self-healing to recover mechanical integrity following deposition [35,36].

Three-dimensional bioprinting enables the fabrication of functional skin substitutes tailored to patient-specific requirements, including the treatment of burn wounds, a significant clinical challenge involving extensive tissue damage, dehydration, and requirements for pro-vascularization biomaterials. Hydrogel-based materials have emerged as promising wound dressings owing to their inherent moisturizing, soothing, and ECM-mimicking properties, which support cell viability and tissue regeneration. Based on these advantages, Fayyazbakhsh et al. [99] investigated 3D-printed hydrogel dressings as ECM-like scaffolds to facilitate cell proliferation and accelerate the healing of deep partial-thickness burn (PTB) wounds. Bioinks composed of varying ratios of gelatin and alginate, incorporating human dermal fibroblasts, were utilized. Among the formulations tested, a porous, patterned dressing containing 75% gelatin and 25% alginate exhibited superior mechanical strength, hydration capacity, and in vitro biological performance, including improved wound closure, hair follicle regeneration, and non-traumatic removal compared with non-printed hydrogel controls. Very recently, Baek et al. [100] established a full-thickness artificial skin model composed of human induced pluripotent stem cell (iPSC)-derived fibroblasts (FBs) and keratinocytes (KCs) within a collagen matrix using an extrusion-based 3D bioprinting system (CLE-iFTs). The proliferation rate of keratinocytes and cell density of the dermal layer in CLE-iFTs were both higher than those of manually fabricated iPSC-derived full-thickness skin models (M-iPSC-FTs), demonstrating the potential of CLE-iFTs as an advanced platform for skin research, disease modeling, and regenerative medicine.

A persistent challenge in skin tissue engineering is the establishment of a functional dermal vascular network to enable permanent engraftment of multilayered skin substitutes and integration with host tissue, particularly for treating chronic, non-healing cutaneous ulcers. Baltazar et al. [101] addressed this limitation by developing an implantable, vascularized, multilayered bioengineered skin graft via 3D bioprinting. A composite bioink comprising human foreskin dermal fibroblasts (FBs), cord blood-derived endothelial colony-forming cells (HECFCs), and placental pericytes (PCs) suspended in rat tail type I collagen was used to form the dermal layer, while a secondary bioink containing human foreskin keratinocytes (KCs) produced the epidermal layer. Keratinocytes proliferated and differentiated to form a stratified epidermal barrier, and endothelial cells and pericytes self-organized into interconnected microvascular networks in vitro, demonstrating the feasibility of generating vascularized skin equivalents.

Silk fibroin (SF)-containing hydrogels are well-suited to extrusion bioprinting for the development of bioactive dressings for burn wound treatment. Indrakumar et al. [102] formulated SF 3D hydrogels via white light-responsive photo-oxidation of tyrosine residues and applied them as bioactive wound dressings for burn wounds. The resulting SF gel-incorporated dressings (SFDs) supported drug loading for local delivery and conformally covered irregular, non-planar burn surfaces. In a subsequent study, a ternary blend ink composed of silk fibroin, poly(vinyl alcohol) (PVA), and methylcellulose was used to fabricate ultra-stretchable hydrogels; following methanol treatment, the 3D-printed constructs exhibited superior mechanical properties, cytocompatibility with human keratinocytes, in vivo biocompatibility in rodent models, and long-term stability suitable for flexible biomedical devices [103]. Kumar et al. [104] reported the development of a polysaccharide-based bioink for direct-write extrusion 3D bioprinting incorporating human skin fibroblasts (CCD-986sk), formulated from sodium alginate combined with carboxylated cellulose nanocrystals (cCNCs) and/or xanthan gum (XG). The inclusion of XG and cCNCs imparted pronounced shear-thinning behavior and contributed to a more interconnected and stable network structure, thereby improving printability. Furthermore, cCNCs enhanced the nanoscale mechanical properties of the hydrogel, including mechanical strength and viscoelastic stiffness. These improvements supported cell adhesion, proliferation, migration, and differentiation, ultimately providing a favorable 3D microenvironment for skin tissue engineering applications [104].

**Figure 5:**
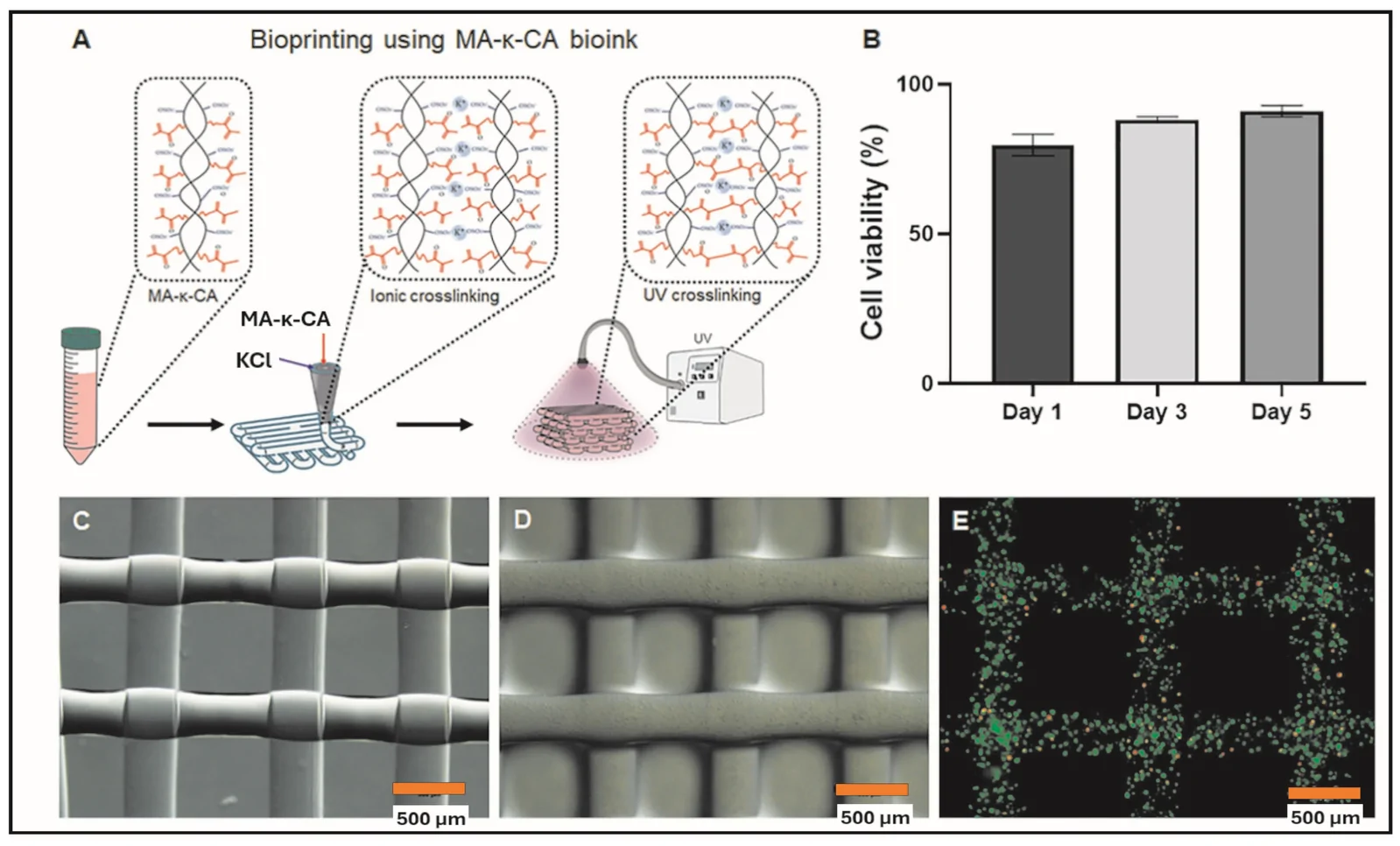
Dual crosslinking (Ionic and UV) mechanism and microscopic images of printed construct: (**A**) Ionic and UV crosslinking mechanisms of cell-laden MA-κ-CA bioink for 3D bioprinting. (**B**) Cell viability assay of 3D-printed cell-laden MA-κ-CA hydrogel. (**C**) Microscopic image of MA-κ-CA hydrogel fiber. (**D**) Microscopic image of encapsulated MA-κ-CA hydrogel fiber at 24 h. (**E**) Live/Dead assay image of 3D-printed MA-κ-CA hydrogel fiber with 2% alginate at day 5 (Scale bars = 500 μm). Reproduced from [106] under the Creative Commons Attribution (CC BY) license.

To further elucidate the cellular responses and underlying molecular mechanisms governing cell fate in fibrous scaffolds, hMSCs were cultured on 3D unidirectionally aligned nanofibers of poly(caprolactone) (PCL) and PCL blended with gelatin (PCL-Gel). These aligned nanofibrous scaffolds enhanced stem cell differentiation, demonstrating the critical role of scaffold topography in directing cell function and lineage commitment [140]. To increase the structural complexity of printed constructs, coaxial 3D bioprinting has emerged as a powerful extension of conventional extrusion, enabling fabrication of multi-material core–shell constructs with independent tuning of mechanical and biological properties. Jergitsch et al. [105] developed a cost-effective, portable coaxial bioprinter to print a soft alginate-gelatin hydrogel core containing MSCs encased within a load-bearing methylcellulose-based shell, maintaining high cell viability while significantly improving shape fidelity over single-material filaments. Lim et al. [106] employed a dual ionic and UV crosslinking strategy for methacrylated κ-carrageenan (MA-κ-CA) bioinks in a coaxial 3D bioprinting system, forming stable NIH/3T3 fibroblast-laden hydrogels with enhanced shape retention and cell compatibility (Figure 5). Similarly, bioinks composed of gelatin methacrylamide (GelMA) and collagen doped with tyrosinase (Ty) were mixed with human melanocytes, keratinocytes, or dermal fibroblasts and 3D bioprinted using a 3D-Bioplotter (Envision Tec, Germany), then crosslinked with UV light (365 nm) to generate cell-laden living skin tissue constructs [107]. Tyrosinase in the bioink served dual roles as a bioactive compound supporting skin regeneration and as an enzyme mediating collagen-GelMA crosslinking; its presence enhanced melanocyte proliferation while significantly inhibiting the growth and migration of dermal fibroblasts.

Three-dimensional freeform fabrication (3D FFF), also referred to as freeform 3D printing or freeform extrusion fabrication (FEF), is an extrusion-based AM approach capable of producing complex, freestanding structures with minimal or no support material. In this context, freeform means the ability to deposit material along nonplanar and spatially unrestricted trajectories, often using multi-axis robotic systems or six-axis printing platforms, rather than being limited to conventional LbL deposition on a flat substrate. Although the material is typically extruded through a nozzle, freeform 3D printing is distinguished not merely by the deposition mechanism but by its capacity to fabricate intricate 3D architectures, including overhanging or suspended features, without relying on conventional supporting scaffolds. Accordingly, the term is often applied to advanced extrusion-based systems that extend beyond standard fused deposition modeling (FDM) or fused filament fabrication (FFF), which generally require layer-wise support for complex geometries. In skin tissue engineering, Lee et al. [108] demonstrated the utility of 3D freeform fabrication for constructing multilayered skin equivalents comprising human dermal fibroblasts and keratinocytes to recapitulate the native organization of skin. Using a robotic and a non-contact dispensing system, collagen hydrogel precursors containing fibroblasts were printed to form the dermal compartment, followed by a keratinocyte-laden collagen layer for the epidermal compartment. Each printed layer was subsequently crosslinked by nebulized aqueous sodium bicarbonate (NaHCO_3_), enabling sequential LbL deposition on a planar tissue-culture surface and yielding bilayered constructs with distinct inner fibroblast and outer keratinocyte regions. To further demonstrate the versatility of this freeform approach for non-planar wound coverage, cell-laden hydrogel constructs were printed directly onto a poly(dimethylsiloxane) (PDMS) mold with 3D surface contours. This strategy demonstrates the potential of freeform cell printing for generating customized skin grafts, as well as engineered tissue models for wound repair, disease modeling, and drug testing.

In the process of wound healing, it is also important to develop tissue scaffolds with antimicrobial resistance that constitute an important functional requirement. Building on strategies for functional scaffold design, bioinspired mechanobactericidal nanostructures were developed using an innovative spin-coating approach to generate nanotopography on the surface of extrusion-based 3D-printed porous polylactide (PLA) scaffolds, based on the principle of polymer demixing. These nanostructured PLA surfaces exhibited excellent bactericidal activity through contact killing of *Pseudomonas aeruginosa* and *Staphylococcus aureus*, while simultaneously supporting the attachment, proliferation, and osteogenic differentiation of pre-osteoblasts [109,110].

Self-healing, granular, or microgel-based bioinks further extend extrusion capabilities. The ideal bioink should simultaneously offer shear-thinning, rapid self-healing, and high structural fidelity, but these properties are difficult to realize in a single homogeneous hydrogel. Zhang et al. [111] mitigated the inherent trade-off between shear-thinning, rapid self-healing, and high structural fidelity by preparing self-healable, pre-crosslinked hydrogel microparticles (pcHµPs) composed of chitosan methacrylate (CHMA) and poly(vinyl alcohol) (PVA). These pcHµPs bioinks displayed excellent shear-thinning during extrusion and rapid self-healing upon shear removal, enabling high-fidelity printing of biomimetic constructs with high aspect ratios and fine structural features. The resulting CHMA/PVA scaffolds supported bone marrow mesenchymal stem cells (BMSCs) growth and spheroid formation, making them attractive candidates for organoid and tissue engineering applications. Overall, standard extrusion bioinks such as gelatin-alginate and silk-PVA blend favor simplicity and clinical scalability. However, they struggle with vascularization and structural complexity. Conversely, coaxial and freeform strategies offer excellent multi-material and non-polar capabilities, but they require high setup complexity and limit manufacturing yield.

### 3.2. Material Jetting/Droplet-Based Bioprinting (DBB)

Material jetting, commonly referred to as droplet-based bioprinting (DBB), is a bioprinting modality in which discrete droplets of bioink are generated and deposited at predefined spatial locations in a non-contact, digitally controlled manner. Unlike extrusion-based bioprinting, which continuously dispenses bioinks through a nozzle under sustained pressure, droplet-based systems eject picoliter- to nanoliter-scale volumes with high spatial resolution, enabling precise positioning of cells, biomolecules, and biomaterials across a substrate [141]. The fundamental operating principle involves the controlled generation of individual droplets from a bioink reservoir, followed by their LbL deposition onto a substrate according to a computer-aided design (CAD) template. Successful droplet formation is governed by a complex interplay of bioink physicochemical properties, including viscosity, surface tension, and elasticity, as well as nozzle geometry and actuation parameters. Because droplet ejection is contactless, DBB provides excellent spatial control over cell placement and enables the fabrication of highly organized, multi-component biological patterns. However, the inherent requirement for low-viscosity bioinks (typically 1–10 mPa∙s) imposes constraints on the mechanical stability of printed constructs, representing a principal limitation relative to extrusion-based approaches, where high-viscosity formulations can provide immediate post-deposition structural support [142,143].

Based on the underlying droplet-generation mechanism, DBB is classified into four major categories, which include inkjet bioprinting, microvalve bioprinting, acoustic bioprinting, and electrohydrodynamic jet (EHDJ) bioprinting. Each modality differs in actuation physics, achievable droplet volume, compatible bioink rheology, and suitability for specific biological applications. Inkjet bioprinting is the most widely used DBB strategy and operates either in continuous jetting or, more commonly in biological applications, in drop-on-demand (DoD) mode. DoD configurations are preferred because they provide precise, on-command control over droplet volume, ejection frequency, and spatial placement. Three principal DoD variants include thermal inkjet, piezoelectric inkjet, and electrostatic inkjet. In thermal inkjet systems, resistive heating elements generate transient vapor bubbles within the ink chamber, and the resulting pressure pulse expels a discrete droplet. In contrast, Piezoelectric systems employ the voltage-driven mechanical deformation of piezoelectric crystals to generate pressure pulses without localized heat generation. Electrostatic inkjet systems apply a voltage differential between an electrode and a pressure plate, inducing electrostatic deflection that transiently alters ink chamber volume and ejects a droplet. Of these, piezoelectric inkjet bioprinting is generally preferred for cell-laden applications, as it avoids thermal stress, minimizes DNA damage, and consistently yields superior cell viability [144,145].

Microvalve bioprinting utilizes a pressurized bioink reservoir coupled to a solenoid- or pneumatically actuated valve, whereby the controlled opening and closing of the valve regulates droplet generation and deposition timing. Compared to conventional inkjet configurations, microvalve systems can accommodate moderately higher bioink viscosities and cell densities, making them compatible with a broader range of hydrogel formulations. These systems have been widely employed for high-throughput tissue engineering and cell-array fabrication [146,147]. Acoustic bioprinting applies focused acoustic waves, typically generated by piezoelectric transducers, to eject droplets from a liquid reservoir without requiring a nozzle. The absence of mechanical shear stress, nozzle contact, and associated clogging risk makes acoustic bioprinting particularly well-suited for bioinks containing mechanosensitive cells, primary stem cells, and biological aggregates such as spheroids. This approach achieves highly accurate droplet placement while maintaining excellent cellular viability and functional integrity [148]. Electrohydrodynamic jet (EHDJ) bioprinting utilizes a high-voltage electric field applied between the nozzle tip and a grounded collector substrate. When the electrostatic force exceeds the surface tension of the bioink meniscus, a Taylor cone forms and emits a continuous or pulsed jet of ultrafine droplets, the diameters of which can be orders of magnitude smaller than those achievable by conventional inkjet systems. This capacity for sub-micron to micron-scale resolution makes EHDJ bioprinting particularly valuable for fabricating microscale biological patterns, spatially defined biomolecule gradients, and intricate tissue interfaces [149] (Table 5).

#### 3.2.1. Bone Tissue Engineering

Inkjet and DoD bioprinting occupy a distinctive niche in bone tissue engineering by enabling non-contact, high-resolution patterning of low-viscosity bioinks with favorable cell viability, capabilities that are especially relevant for organotypic hydrogel constructs demanding precise spatial organization of multiple cell types and osteoinductive biomolecules. Because these systems are constrained to bioinks of relatively low viscosity, printed constructs typically require post-deposition crosslinking strategies, including ionic, photochemical, or enzymatic gelation, to achieve adequate structural stability for load-bearing applications. Within these constraints, inkjet and DoD platforms have demonstrated considerable utility in regenerative medicine, particularly where spatial fidelity and cell preservation are prioritized over immediate mechanical performance (Table 5).

Blaeser et al. [152] introduced a microvalve-based bioprinting approach to fabricate multimaterial 3D constructs with high spatial resolution, demonstrating that the shear stress sustained by cells during valve actuation was precisely controlled to maintain post-printing viability. Operating on the principle that hydrogel composition profoundly influences cellular response, Duarte Campos et al. [150] employed inkjet-based bioprinting to produce mesenchymal stem cell (MSC)-laden bone tissue substitutes from agarose-collagen blend hydrogels. In this system, agarose provided printability and structural stiffness, while collagen provided biological adhesion motifs and matrix cues that promoted cell spreading. Notably, increasing collagen content progressively altered MSC morphology, enhanced cytoskeletal organization, and directed osteogenic differentiation, emphasizing the importance of hydrogel compositional tuning in guiding lineage commitment. Such MSC-laden bioprinted constructs have subsequently been proposed as platforms for non-viral gene delivery, localized incorporation of osteogenic growth factors (e.g., BMP-2, TGF-β), and co-culture with endothelial progenitor cells to promote construct vascularization [150].

The preservation of thermally labile bioactive molecules during fabrication represents an additional advantage of low-temperature DBB approaches. Inzana et al. [151] demonstrated this principle through an inkjet-based 3D printing process in which calcium phosphate powder was bound using phosphoric acid solutions supplemented with type I collagen. During printing, acid-mediated dissolution and subsequent reprecipitation of calcium phosphate produced collagen-calcium phosphate composite scaffolds with interconnected porosity and tunable mechanical properties. When combined with osteoinductive factors, these constructs significantly enhanced bone healing in critical-size defect models, establishing a robust framework for mineral-organic composite fabrication via inkjet-based additive manufacturing.

Hierarchical silk-bioactive glass (SF-BG) composites have also been successfully engineered through the synergistic combination of indirect inkjet 3D printing with freeze-drying techniques to create architecturally complex scaffolds with tailored biological functionality. Bidgoli et al. [154] fabricated 3D bioactive SF-BG scaffolds incorporating nano- (<100 nm) or micro-scale (~6 µm) 45S5 glass particles, achieving compressive strengths of 0.94 (nano) and 1.2 MPa (micro) under dry conditions. Nanoparticle-reinforced scaffolds exhibited superior hBMSCs adhesion and significantly elevated ALP activity by day 14, reflecting pronounced osteoinductive potential for load-bearing bone regeneration.

Therefore, inkjet and DoD systems achieve excellent resolution and high cell survival rates, but they diminish mechanical robustness, which necessitates that the constructs require secondary crosslinking methods [150,152], a workaround rather than a solution to load-bearing applicability.

#### 3.2.2. Skin Tissue Engineering

Droplet-based precision dispensing, a closely related DBB strategy, involves the highly controlled deposition of liquid droplets onto a substrate in an LbL fashion. Classified broadly as a form of material jetting or DoD printing, this approach enables high-resolution, multi-material fabrication with minimal material waste and low risk of cross-contamination between ink reservoirs [155]. For biological applications targeting skin regeneration, collagen is the predominant hydrogel candidate given its natural abundance in the dermal ECM and its well-established role in supporting keratinocyte and fibroblast function. However, the inherent low viscosity, slow gelation kinetics, and tendency toward structural collapse during and after deposition make collagen challenging to bioprint without formation of modification. To address these limitations, Shafiee et al. [153] demonstrated that vapor-phase ammonia crosslinking applied in situ during droplet deposition could rapidly neutralize the acidic collagen solution, triggering gelation and stabilizing printed filaments while preserving encapsulated cell viability, a strategy that effectively decouples printability requirements from post-deposition stabilization.

Beyond material properties, the mechanical stresses inherent to nozzle-based bioprinting systems represent an important physiological factor, as shear and impact forces during droplet ejection and substrate contact potentially compromise cell membrane integrity and downstream function. Thaden et al. [156] investigated these biophysical effects using giant unilamellar vesicles (GUVs) as synthetic membrane models in both DoD and extrusion-based bioprinting configurations. Their findings revealed that approximately 65% of GUVs remained structurally intact at a dispensing pressure of 0.5 bar, with membrane rupture increasing at higher actuation pressures. Supplementation of the dispersion medium with PEG enhanced GUV stability through steric interactions with the lipid bilayer, while this modification diminished certain membrane-mediated interactions with human cells post-printing. Significantly, GUVs loaded with chlorin e6-PEG conjugates and fluorescent cargo released their payload upon light illumination, demonstrating a proof-of-concept for optically triggered, on-demand intraconstruct delivery of growth factors, drugs, nutrients, or signaling gases at millimeter-to-centimeter spatial scales. These results emphasize the potential of stimuli-responsive engineered vesicle systems as functional payload-delivery modules within bioprinted skin and soft tissue constructs.

### 3.3. Laser-Mediated and Light-Assisted Bioprinting

**Figure 6:**
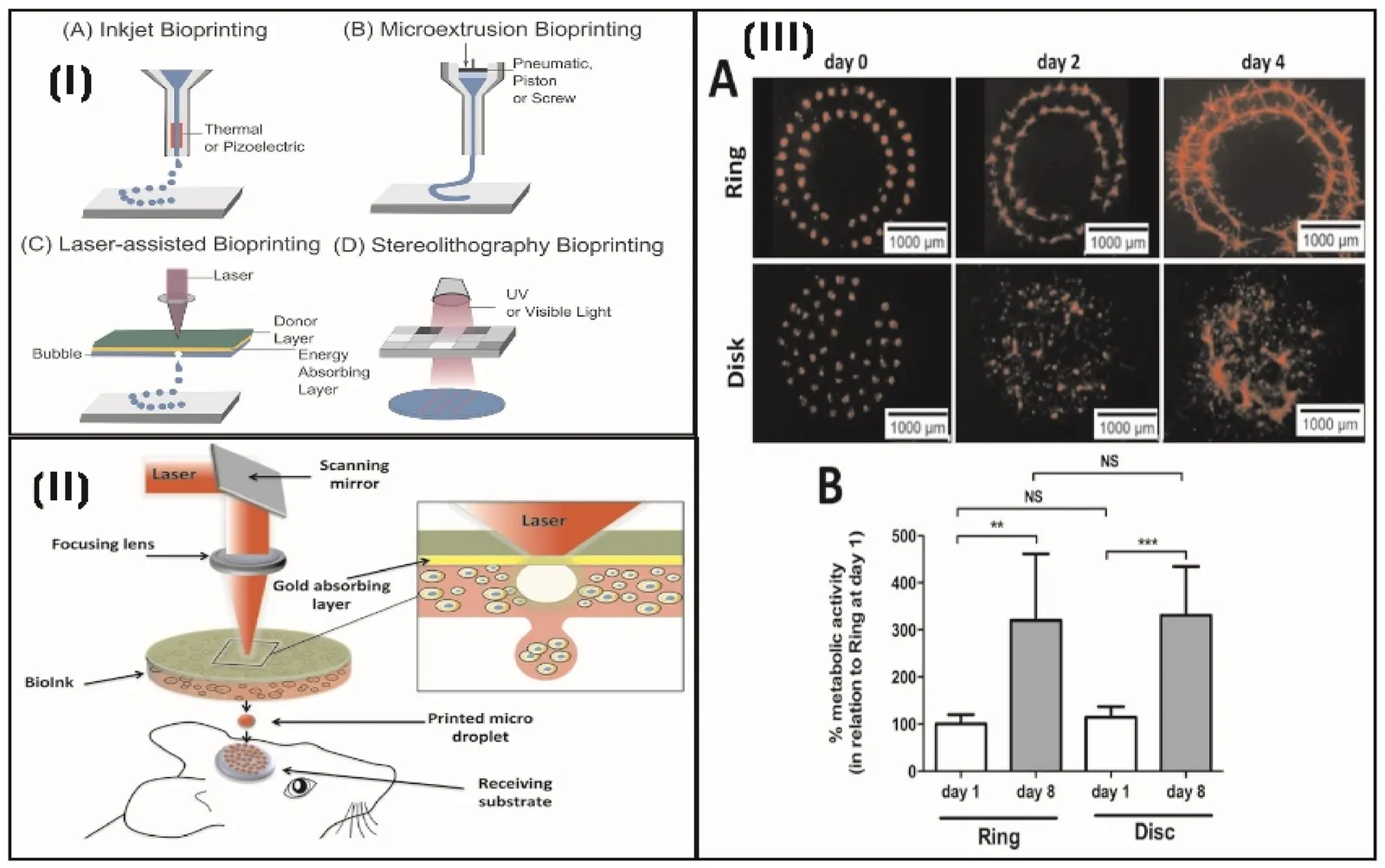
(**I**) Examples of different bioprinting methods: (**A**) Inkjet bioprinters deposit small droplets of hydrogel and cells to build tissue layer-by-layer. (**B**) Microextrusion bioprinters deposit a cell-laden liquid solution via pneumatic or manual force. (**C**) Laser-assisted bioprinting uses a laser to rapidly heat a donor layer (green), which forms a bubble propelling the bioink onto the substrate. (**D**) Stereolithography bioprinters use UV or visible light to selectively cross-link bioinks layer by layer to build a 3D construct. Reproduced with permission. [25]. Copyright (2018), Wiley-VCH GmbH. (**II**) Schematic representation of the laser-assisted bioprinting (LAB) approach. A typical LAB setup comprises a pulsed laser beam, a focusing system, a ribbon (a transparent glass slide, coated with a laser-absorbing layer of metal, onto which a thin layer of bioink is spread, and a receiving substrate facing the ribbon. The physical principle of LAB is based on the generation of a cavitation-like bubble into the depth of the bioink film, whose expansion and collapse induce the formation of a jet and, thereby, the transfer of the bioink from the ribbon to the substrate (here, a bone defect on the mouse calvaria), forming a microdroplet. (**III**) (**A**) Representative fluorescence images of ring and disk printed tomato-positive D1 cells at days 0, 2 and 4. (**B**) Percent metabolic activity, as measured by the resazurin assay, of D1 cells printed in a ring or disk geometry at days 1 and 8, in relation to ring geometry at day 1 (Average ± SD, *n* = 6, ** and *** denotes *p* < 0.01 and *p* < 0.001, respectively). Reproduced from [157], licensed under CC BY 4.0.

Laser-mediated bioprinting (LMB) represents a high-resolution, nozzle-free biofabrication technology that utilizes laser energy to precisely deposit living cells, biomaterials, and bioactive molecules into predefined 3D architectures [157] (Figure 6). Unlike extrusion- and droplet-based bioprinting, LMB eliminates direct mechanical contact between the printing device and bioink, thereby avoiding nozzle clogging and reducing shear-induced cellular damage. Owing to their exceptional spatial accuracy and ability to process a wide range of viscosities, laser-based techniques have become valuable tools for engineering complex tissues, vascular networks, neural constructs, and microphysiological systems [23]. LMB involves the conversion of optical energy into a localized mechanical force capable of transferring biomaterials from a donor substrate to a receiving surface. Depending on the mechanism of energy transfer and material deposition, LMB is classified into three categories, namely laser-induced forward transfer (LIFT), matrix-assisted pulsed laser evaporation direct writing (MAPLE-DW), and laser-guided direct writing (LGDW) [158,159].

The LIFT technique uses a pulsed laser beam focused onto an energy-absorbing layer coated beneath a thin film of bioink. Laser irradiation generates a rapidly expanding vapor bubble that propels a microdroplet of bioink toward a receiving substrate. LIFT typically achieves a resolution below 100 µm while maintaining cell viability exceeding 90%. LIFT is suitable for patterning stem cells, endothelial cells, and multicellular tissue interfaces requiring precise spatial organization [160,161]. MAPLE-DW evolved from thin-film deposition technologies and utilizes laser pulses to transfer biomaterials embedded within a sacrificial matrix onto a target substrate. The matrix absorbs laser energy and facilitates material ejection while minimizing direct laser exposure to biological components. This approach enables the controlled deposition of proteins, growth factors, and biomaterials alongside living cells with high positional accuracy. Although the printing speed is much lower than that of extrusion-based methods, this technique offers superior control over cellular distribution and biomolecular patterning [162]. In laser-guided direct writing (LGDW), optical forces generated by a focused laser beam trap and guide individual cells or cell clusters toward specific locations on a substrate. This method is similar to optical tweezers, allowing non-contact manipulation of living cells with high precision. It is utilized in constructing microscale biological architectures where single-cell positioning is required [163,164].

Similarly, light-assisted 3D bioprinting (LAB), also known as vat-photopolymerization bioprinting, is a rapidly advancing biofabrication technology that utilizes spatially controlled light exposure to selectively polymerize photosensitive bioresins into 3D biological structures. Unlike extrusion- and droplet-based bioprinting techniques, which depend on the physical deposition of biomaterials, light-assisted bioprinting fabricates constructs through the photochemical crosslinking of bioinks containing photopolymerizable polymers, cells, and bioactive molecules. This approach offers exceptional spatial resolution, rapid fabrication speeds, smooth surface morphology, and the ability to generate highly complex geometries that closely mimic native tissue architectures. Consequently, light-based bioprinting has become increasingly important for tissue engineering, regenerative medicine, organoid fabrication, microphysiological systems, and organ-on-chip technologies [165]. LAB involves exposing a photosensitive bioresin to a light source of a specific wavelength, typically ultraviolet (UV) or visible light. Upon illumination, photoinitiators generate reactive species that induce polymer crosslinking, transforming liquid bioresins into solid hydrogel structures. The fabrication process occurs through point-by-point scanning, LbL projection, or volumetric photopolymerization, depending on the printing modality. Compared with nozzle-based systems, LAB provides superior resolution and geometric fidelity while minimizing mechanical stresses on encapsulated cells. However, parameters need to be optimized for photoinitiator concentration, light dosage, exposure time, and bioresin composition to avoid phototoxicity and preserve cellular functionality [166]. LAB is categorized into stereolithography (SLA), digital light processing (DLP), two-photon polymerization (TPP), and volumetric bioprinting (VBP).

Stereolithography (SLA) is the earliest light-based additive manufacturing technology and forms the foundation of modern bioprinting. In SLA, a focused laser beam scans across the surface of a photosensitive bioresin, selectively curing predefined regions LbL. The build platform then moves vertically, allowing subsequent layers to be crosslinked until the final construct is completely fabricated. SLA offers high precision and excellent structural fidelity, but is generally slower because each layer must be individually scanned. Nevertheless, SLA remains highly valuable for fabricating vascularized tissues, cartilage constructs, and hydrogel-based scaffolds [167]. Digital light processing (DLP) bioprinting utilizes a digital micromirror device (DMD) to project an entire 2D image onto the bioresin simultaneously. Consequently, complete layers are polymerized in a single exposure rather than through sequential laser scanning. This significantly increases fabrication speed while maintaining excellent resolution. DLP systems have become one of the most widely used light-assisted bioprinting platforms due to their high throughput, reproducibility, and ability to generate complex tissue constructs with microscale precision [168]. Two-photon polymerization (TPP), also known as multiphoton lithography, is an advanced laser-based technique capable of producing structures with submicron and even nanometer-scale resolution. In TPP, a femtosecond laser induces simultaneous absorption of two photons within a highly localized focal volume, initiating polymerization exclusively at the focal point. This nonlinear optical phenomenon enables 3D fabrication without strict LbL processing. TPP is particularly useful for creating biomimetic extracellular matrix (ECM) architectures, microvascular networks, and cellular guidance structures [169]. Volumetric bioprinting (VBP) represents the next generation of light-assisted biofabrication technology. Instead of constructing structures LbL, VBP polymerizes entire 3D objects within a rotating volume of photosensitive bioresin through tomographic light projection. This approach enables the fabrication of centimeter-scale, cell-laden constructs within seconds to minutes while maintaining high cellular viability. The ability to rapidly generate anatomically complex tissues without layer interfaces has positioned volumetric bioprinting as a promising platform for future organ-scale biofabrication [170]. Because all of these modalities depend on photopolymerizable or photoresponsive bioinks, recent advancements in bioink engineering, particularly the methacrylation of natural polymers such as silk fibroin, gelatin, and κ-carrageenan, emphasize tuning the degree of functionalization, photoinitiator chemistry, and light dose to balance printability, mechanical fidelity, and cytocompatibility. The following discussion details the advances in the applications of DLP- and LaBP-based bioprinting across bone, cartilage, skin, and vascular tissue engineering (Table 6).

#### 3.3.1. Bone Tissue Engineering

Advancing these strategies, Rajput et al. [171] synthesized photocurable methacrylated silk fibroin (SF-MA, 67.3% degree of methacrylation) specifically tailored for DLP bioprinting. The SF-MA bioink supported high-precision printing of complex 3D hydrogel architectures, including trabecular- and Haversian-bone-mimetic geometries, in which encapsulated pre-osteoblasts remained highly viable and exhibited robust osteogenesis, with cell-mediated calcium deposition increasing progressively over 14 days, demonstrating the bioink’s suitability for bone tissue engineering. Extending this approach, Waidi et al. [172] developed a composite DLP bioink combining photocurable silk fibroin with bacterial nanocellulose (silk-MA/BNC). MC3T3-E1 pre-osteoblasts immobilized within silk-MA/BNC scaffolds containing up to 0.75 wt% BNC showed enhanced biomineralization, calcium deposition, and improved cell viability and metabolic activity relative to unreinforced silk-MA, demonstrating the advantages of nanocellulose reinforcement in DLP-printed bone constructs. To impart osteogenic and antibacterial properties, Kumari et al. [173] incorporated bioactive silica nanoparticles (BSNPs) in methacrylated κ-carrageenan (MA-κ-CA), generating MA-κ-CA-BSNP composite hydrogels. DLP-printed MC3T3-E1 pre-osteoblast-laden MA-κ-CA-BSNP scaffolds exhibited high cell viability, no cytotoxicity, and enhanced osteogenic differentiation over 21 days in vitro, while a 21-day in vivo study in Wistar rats revealed no inflammatory response, confirming the scaffolds’ biocompatibility and suitability for bone tissue engineering.

Photopatternable hydrogels more broadly offer spatiotemporal control over cell distribution and microscale 3D geometry and are increasingly used in advanced 3D culture systems [186]. However, conventional layer-by-layer (LbL) photolithography, stereolithography, and direct-write printing approaches struggle to produce curved, anatomically accurate, micropatterned, cell-laden geometries. Self-folding, or bio-origami, fabrication, built from biological materials such as proteins and DNA, addresses this challenge by transforming planar hydrogel films into curved 3D geometries through programmed internal strains [187,188]. Jamal et al. [174] described the self-folding of photopatterned polyethylene glycol (PEG)-based hydrogel bilayers, composed of polymers with differing molecular weights, into curved, anatomically relevant micrometer-scale geometries that supported long-term viability of insulin-secreting β-TC-6 cells and sustained insulin release. These self-folding bio-origami constructs were further used as in vitro models of ductal carcinoma. Kwag et al. [175] encapsulated MDA-MB-231 human breast adenocarcinoma cells within curved and tubular photopatterned PEG-diacrylate (PEGDA) bilayer hydrogels using a self-folding approach that mimics mammary ducts and acini; incorporating methacrylated gelatin (methagel) into the PEGDA bilayer enhanced cell adhesion and spreading.

#### 3.3.2. Cartilage Tissue Engineering

Digital light processing (DLP) bioprinting enables rapid, layer-by-layer (LbL) photopolymerization of photocurable bioinks using projected light patterns, offering high print speed and fine feature resolution for fabricating complex 3D architectures. When combined with biocompatible, biodegradable, and light-responsive bioinks, DLP platforms can generate cell-laden constructs that closely reproduce the microarchitecture and mechanical behavior of native tissues [176,177]. As in bone tissue engineering, methacrylated silk fibroin has proven an attractive DLP bioink for cartilage and other organ-like constructs. Kim et al. [176] synthesized glycidyl methacrylate-functionalized silk fibroin (Sil-MA), yielding a structurally stable and biocompatible bioink that enabled the fabrication of highly complex heart-, vessel-, brain-, trachea-, and ear-like structures. Building on this platform, Hong et al. [177] prepared glycidyl methacrylate-modified silk fibroin (Silk-GMA) for DLP printing of chondrocyte-laden hydrogels; in vitro, silk-GMA constructs supported chondrocyte viability, proliferation, and differentiation over four weeks of culture, while implantation in a partially defected rabbit trachea model showed formation of cartilage-like tissue and a surrounding epithelium in vivo.

3D printing with a DLP printer has ushered in a new wave in tissue engineering and regenerative medicine when used with biocompatible, biodegradable, and printable bioinks. It is also a rapid fabrication technique for producing complex 3D cell-laden scaffolds for tissue engineering applications. Photoinitiated crosslinking has also been optimized for collagen-rich bioinks. Because collagen exhibits slow gelation, limited printability, and poor mechanical integrity, hybrid bioinks combining collagen with synthetic polymers, as well as strategies for depositing collagen into sacrificial support hydrogels, have been developed to improve structural fidelity and handling during bioprinting [94,189]. To further enhance the mechanical properties of collagen-based constructs, Tirella et al. [178] introduced a photo-crosslinking strategy in which riboflavin-mediated radical polymerization, initiated by UV irradiation, was used to tailor collagen scaffolds for soft tissue-mimetic applications. Subsequently, in an extrusion-printed bioink that was subsequently stabilized by secondary photocrosslinking, Diamantides et al. [179] demonstrated that blue-light-activated riboflavin crosslinking improved the storage modulus and gelation kinetics of type I collagen bioinks in a strongly pH-dependent manner, with maximal gel modulus and print shape fidelity observed between 8 and 9.5. Although riboflavin crosslinking increased printability and mechanical stability, it also reduced bovine chondrocyte viability following blue-light exposure, where pH itself did not significantly affect cell viability. These developments complement click-chemistry hydrogels and visible-light photoinitiator systems that continue to expand the range of capabilities for DLP and other photopatterning-based bioprinting approaches.

#### 3.3.3. Skin Tissue Engineering

Laser-assisted bioprinting (LaBP) is a high-resolution, nozzle-free 3D printing technique that uses laser-induced forward transfer (LIFT) to precisely deposit living cells and biomaterials, generally achieving higher cell viability and finer spatial precision than nozzle-based extrusion techniques [166]. In regenerative medicine, the precise spatial organization of living cells within 3D microenvironments that recapitulate native extracellular matrix architecture is essential for developing functional tissue substitutes. LaBP enables deposition of highly concentrated cell suspensions with micrometer-scale precision, allowing picoliter- to femtoliter-scale droplets containing cell densities of up to 10^8^ cells/mL to be patterned into predefined 3D architectures. Gruene et al. [180,181] and Koch et al. [182] demonstrated the capability of LaBP to spatially organize cells in 3D configurations, and Koch et al. [183] subsequently reported the first 3D bioprinting of dermal fibroblasts and keratinocytes embedded in collagen to generate a native-like skin substitute. The printed skin constructs established intercellular adhesion and communication through adherens and gap junctions, demonstrating the potential of LaBP for the fabrication of autologous skin grafts and other engineered tissue substitutes.

Polysaccharide-based photocurable systems have also been developed for DLP-based skin constructs. Kumari et al. [184] synthesized methacrylated κ-carrageenan (MA-κ-CA) as a visible-light-curable bioink and used 405 nm light to produce cell-laden hydrogels with intricate architectures through LbL DLP printing. NIH/3T3 fibroblasts encapsulated in MA-κ-CA constructs retained high viability and proliferative capacity over several days, mimicking features of native tissue complexity. Together, these laser- and light-based strategies demonstrate complementary routes to reconstructing the layered architecture of native skin, which include single-cell-resolution LIFT patterning versus bulk photopolymerization of a pre-formed bioink.

#### 3.3.4. Vascular Tissue Engineering

Achieving functional vascularization remains a critical limitation in the fabrication of thick, biomimetic tissue constructs, as diffusion-mediated oxygen and nutrient transport is restricted to approximately 100–200 µm, beyond which hypoxia-induced necrosis occurs [190,191]. To maintain parenchymal cell viability, bioprinted tissues require a fundamental shift from passive diffusion to active perfusion through perfusable, hierarchically organized vascular networks that remain structurally resilient under physiological hemodynamic forces, including physiological shear stress and pulsatile flow [192]. However, hollow lumen patency (the open, unblocked space inside a blood vessel) is insufficient to establish functional performance. It requires the establishment of a fully mature endothelium, wherein endothelial cells form a continuous, non-thrombogenic barrier characterized by robust tight junctions, regulated permeability, and vasoactive responsiveness. This maturation process is further dependent upon the recruitment and integration of mural cells, such as pericytes and smooth muscle cells, which are essential for vascular stabilization, remodeling, and long-term functionality [193]. In addition, effective host integration requires rapid and efficient inosculation between the engineered vasculature and the host microcirculatory system to mitigate ischemic injury immediately following implantation [194]. Despite significant advances, current bioprinting technologies remain fundamentally limited by an inherent limitation between printing resolution and construct scale, limiting their ability to integrate macrovascular conduits with capillary-scale networks [195]. Therefore, the coordinated resolution of challenges related to perfusion dynamics, endothelial and mural cell maturation, and surgical anastomotic integration is critical for the successful translation of macroscopic, multi-layered engineered tissues into functional tissue replacements.

Nature-inspired self-forming strategies have also been explored to generate tubular vascular structures. One such approach uses stress-induced rolling of electrospun poly(lactic-co-glycolic acid)/polycaprolactone (PLGA/PCL) mats to produce hollow microvessel scaffolds, inspired by the curling of dried apple peels into tubules. Within the light-based bioprinting domain specifically, Zhang et al. [185] fabricated micro-scaled hollow tubules (MHTs) by photocrosslinking and curling planar gelatin methacrylamide (GelMA) hydrogel layers, exploiting differential contractility and swelling between the upper and lower faces of each sheet. By tuning photoinitiator (Irgacure) concentration and UV exposure dose, robust, reproducible 3D shape-morphing was achieved at the microscale, yielding tubules 50–600 µm in diameter with good tensile strength, endothelial cytocompatibility with seeded HUVECs, and the capacity to promote neovascularization in vivo in a rat flap model. This self-folding principle, transforming a 2D photopolymerized sheet into a curved 3D geometry via programmed internal strain, is mechanistically continuous with the bio-origami strategies, demonstrating that a singular material and lithographic platform can be redirected across diverse target tissues simply by adjusting bilayer geometry and crosslink density.

## 4. Scaffold-Free Bioprinting and Bioassembly in Tissue Engineering and Regenerative Medicine

Scaffold-free bioprinting and bioassembly are cell-centric fabrication strategies in which living cellular aggregates, rather than cell-laden hydrogel inks or prefabricated scaffolds, serve as the primary structural units for tissue construction. Tissue engineering approaches have been classified into scaffold-based and scaffold-free strategies. Among these, scaffold-free 3D bioprinting has gained significant importance by utilizing organ building blocks (OBBs) capable of self-assembly and self-organization, such as tissue spheroids, organoids, and assembloids. In this approach, multicellular building blocks are assembled into predefined geometries and subsequently mature through biological self-organization, aggregate fusion, endogenous ECM deposition, and tissue remodeling [196]. 3D bioprinting techniques include extrusion-based, jetting-based, and vat photopolymerization-based methods. Meanwhile, bioassembly techniques utilize the Kenzan method, fluid-based manipulation, microfluidics, bioprinting-assisted tissue formation, and aspiration-assisted technologies. While scaffold-based strategies focus primarily on constructing physical frameworks to mimic the native ECM, scaffold-free strategies emphasize the assembly of cellular building blocks, utilizing intrinsic cell–cell adhesion, endogenous ECM production, and developmental morphogenetic processes to generate cohesive tissue structures [197].

Multicellular spheroids remain the most widely used building blocks in scaffold-free biofabrication. Typically containing hundreds to thousands of cells, these spheroids are generated using low-adhesion culture plates, hanging-drop systems, rotary culture, centrifugation-assisted aggregation, microfluidic droplets, or microwell arrays [198]. During this process, spheroids develop tissue-like features, including direct cell–cell communication, endogenous matrix deposition, oxygen and nutrient gradients, and cell-type-dependent spatial organization, making them more physiologically accurate than 2D cultures and highly concentrated in cellular content compared to conventional hydrogel systems [199]. The biological foundation of scaffold-free bioassembly is closely related to tissue fusion and cell-sorting phenomena observed during embryonic development, morphogenesis, and wound repair. When spheroids are placed in close contact, adjacent aggregates fuse through cell migration across the contact interface, cytoskeletal contraction, ECM remodeling, and the progressive minimization of interfacial surface energy. Fusion kinetics depend strongly on spheroid size, cell type, ECM composition, maturation state, and culture conditions. Smaller spheroids, typically ranging from 300 to 500 µm, are preferred due to ease of manipulation, superior cell viability, efficient nutrient diffusion, and rapid fusion kinetics [200,201]. Conversely, larger aggregates provide greater cellular mass but are highly susceptible to hypoxia, necrotic core formation, and delayed integration due to limited oxygen and nutrient transport.

Scaffold-free bioassembly offers several distinct advantages for tissue engineering and regenerative medicine. It enables the generation of constructs with very high initial cell densities that closely approach native tissue cellularity, while avoiding potential foreign-body responses associated with non-degradable or slowly degrading synthetic polymers. Because the final matrix is entirely produced by the constituent cells, the resulting tissue exhibits enhanced biological remodeling and tissue-specific functional maturation [202]. Notably, stem cell spheroids preserve viability and paracrine activity more effectively than dispersed cells, demonstrating enhanced angiogenic, anti-inflammatory, antifibrotic, and immunomodulatory functions critical for regenerative therapies [203]. Nevertheless, scaffold-free constructs present notable limitations, including slow fusion kinetics, limited early mechanical stability, difficulty in fabricating large-scale constructs, diffusion constraints, and the requirement for prolonged in vitro maturation prior to implantation [204].

### 4.1. Spheroid-Based Bioprinting and Bottom-Up Tissue Assembly

Spheroid-based bioprinting is a scaffold-free or scaffold-minimized bioassembly method in which preformed multicellular spheroids are used as “living building blocks” to construct macroscale tissue architectures. The process begins with controlled spheroid generation, followed by precise spatial placement into designed patterns such as sheets, rings, tubes, vascular conduits, patches, or layered structures. Following deposition, the spheroids fuse into continuous tissues through cell-mediated remodeling rather than being immediately fixed by a permanent hydrogel network. Previously, Norotte et al. [198] demonstrated that multicellular spheroids and tissue cylinders were assembled into scaffold-free, vascular-like structures, establishing the feasibility of using cellular aggregates as bioink-like particles for organ printing. Similarly, Jakab et al. [205] utilized multicellular spheroids as “bio-ink” particles deposited into a supportive “bio-paper” environment, showing that cell aggregates self-assemble into living structures with defined shapes. When applied to cardiac constructs composed of embryonic cardiac and endothelial cells, this strategy produced synchronously beating tissue blocks with early endothelial organization into vessel-like structures, demonstrating the potential of spheroid fusion for functional tissue fabrication.

**Figure 7:**
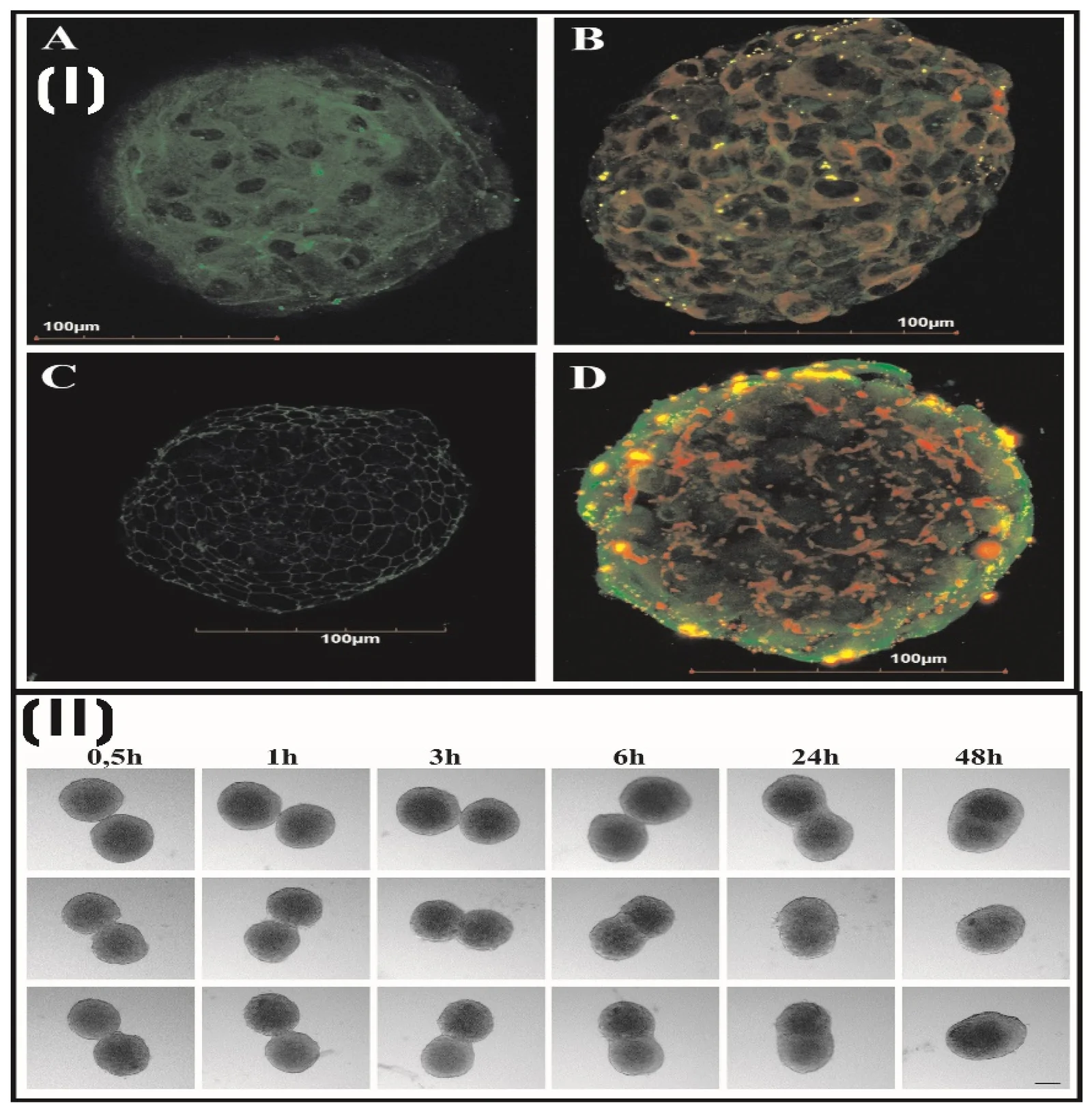
(**I**) Expression of markers in seven-day spheroids from the L-MSCs (**A**,**B**) and RPE cells (**C**,**D**): (**A**) Vimentin; (**B**) laminin in the cytoplasm (red) and nestin (green) in a few cells. (**C**) Tight junctions with an expression of ZO-1; (**D**) laminin in the cytoplasm and between cells (red) and nestin (green) in the cells of a surface layer. Laser scanning confocal microscopy. (**II**) The dynamics of spheroid fusion within 48 h in a hanging drop system. Two L-MSC spheroids (upper row), two RPE-cell spheroids (middle row), and L-MSCs with RPE-cell spheroids (lower row). Scale bar: 100 µm. Reproduced from [206], licensed under CC BY 4.0.

However, spheroid fusion kinetics are not uniform across cell types or building-block architectures. Kosheleva et al. [206] investigated the fusion behavior of spheroids derived from limbal mesenchymal stem cells and retinal pigment epithelial cells, showing that epithelial spheroids fused more rapidly, a behavior associated with lower apparent surface tension and distinct ECM composition (Figure 7). More recently, Kopinski-Grunwald et al. [207] reported that scaffolded spheroids (S-SPHs) exhibited stable and robust fusiogenicity independent of their maturation state, suggesting that microscale internal supports may improve the reproducibility of spheroid-based bioassembly while retaining the advantages of bottom-up tissue construction. The applications of spheroid-based bioprinting include vascular grafts, cardiac patches, cartilage constructs, bone-like tissues, liver models, tumor models, and skin equivalents. Endothelial and smooth muscle cell spheroids can be arranged to generate multilayered vessel analogs, whereas dermal and epidermal spheroids can be assembled into stratified skin-like tissues. In cancer research, tumor spheroids are widely utilized to recapitulate cell–cell interactions, hypoxia gradients, drug penetration barriers, and therapeutic resistance. In regenerative medicine, mesenchymal stem cell (MSC) spheroids are particularly attractive because their enhanced paracrine activity improves angiogenesis, immunomodulation, and the repair of ischemic, cutaneous, musculoskeletal, and inflammatory tissue injuries [205,208,209]. Together, these principles establish spheroids as modular living building blocks for scaffold-free tissue construction. To organize these aggregates into predefined architectures, several spheroid-positioning and bioassembly strategies have been developed. These include microneedle array bioprinting (the Kenzan method), aspiration-assisted bioprinting, magnetic field-directed bioassembly, acoustic and dielectrophoretic bioassembly, droplet-based spheroid deposition, and microfluidic bioassembly. Each of these platforms offers distinct trade-offs between spatial precision, throughput, and compatibility with different spheroid sizes and cell types.

#### 4.1.1. Microneedle Array Bioprinting (The Kenzan Method)

The Kenzan method is one of the most established scaffold-free, mechanical pick-and-place bioprinting strategies. This robotic platform arranges multicellular spheroids into predesigned contiguous structures with micron-level precision, using stainless steel microneedles (Kenzans) as temporary support while the constructs are cultivated until they fuse into a coherent tissue. This method was invented by Professor Koichi Nakayama at Saga University, who focused on the intrinsic self-aggregation features of cells to assemble spheroids into 3D macroscopic tissues without the need for exogenous collagen or hydrogel matrices [33,210]. A commercial realization of this technology is the automated “Regenova” Bio-3D printing system by Cyfuse Biomedical K.K. (Tokyo, Japan), which enables the high-precision fabrication of 3D cellular structures [211]. During this process, spheroids are robotically picked and impaled sequentially onto the Kenzan needle array according to a computer-designed (CAD) model. The microneedle array temporarily maintains the structural geometry during the initial fusion phase. After sufficient in vitro maturation, the fused tissue is gently removed from the needles and further cultured to promote endogenous matrix deposition, tissue remodeling, and mechanical strengthening.

Mechanistically, the needle array itself is precisely engineered to balance optimal spheroid retention against potential tissue damage. The microneedle array typically consists of stainless steel needles with a diameter of 100–200 µm and a pitch of 300–500 µm. A customized nozzle arranges the preformed spheroids sequentially, stacking them on top of one another within each needle according to the predetermined architecture. Spheroid diameters of approximately 300–500 µm are commonly used because they can be manipulated reproducibly while maintaining adequate nutrient diffusion and cell viability [148,212]. A key advantage of the Kenzan method is that it avoids embedding cells within a bulk exogenous hydrogel. In contrast to material-dependent and energy-intensive LbL deposition approaches such as inkjet, microextrusion, or laser-assisted bioprinting, this scaffold-free bioassembly facilitates direct cell–cell contact, endogenous ECM formation, and tissue maturation under conditions that mimic embryonic and developmental self-organization [32].

The Kenzan method has been widely applied to fabricate vascular grafts, cartilage-like tissues, cardiac patches, neural constructs, tracheal tissue, and biomimetic skin models. For instance, Machino et al. [213] demonstrated the fabrication of scaffold-free trachea-like tubes to replace the epithelium and capillaries in rat tracheas by precisely inserting spheroids into a microneedle array using a Bio-3D printing system. In vascular tissue engineering, spheroids containing endothelial cells, smooth muscle cells, and fibroblasts are organized into tubular structures that mature into scaffold-free vessel-like constructs [214]. Similarly, for cartilage repair, chondrocyte or mesenchymal stem cell (MSC) spheroids were assembled into mechanically coherent tissues characterized by robust chondrogenic matrix deposition [215]. In cardiac applications, spheroids containing cardiomyocytes and supporting stromal or endothelial cells were patterned to generate contractile patches or microtissues [216]. Collectively, these examples demonstrate the ability of the Kenzan approach to produce dense, cell-rich tissues with minimal foreign material.

Despite these advantages, the Kenzan method presents several limitations. The microneedles can cause mechanical damage to the spheroids during impalement, and removing the mature construct from the needle array requires sufficient tissue cohesion to prevent tearing or deformation. Furthermore, fabrication speed is strictly constrained by the sequential, single-spheroid placement mechanism, which limits throughput for macroscale constructs. To overcome this limitation, Kim and colleagues developed HITS-Bio (High-throughput Integrated Tissue Fabrication System for Bioprinting), a multi-array bioprinting platform that utilizes a digitally controlled nozzle array (DCNA) to place spheroids ten times faster than conventional single-nozzle systems while maintaining greater than 90% cell viability. This system enables rapid, scalable tissue engineering, successfully achieving calvarial bone regeneration (~30 mm^3^) in a rat model and producing 1 cm^3^ cartilage constructs within 40 min [217]. Beyond throughput and mechanical injury constraints, early-stage Kenzan-fabricated constructs lack immediate structural stability, requiring extended culture prior to surgical manipulation. Additionally, vascularization remains a major challenge for thick tissues, as diffusion limitations can compromise core cell viability unless perfusable channels or prevascular networks are actively integrated.

#### 4.1.2. Aspiration-Assisted Bioprinting (AAB)

Aspiration-assisted bioprinting (AAB) is a nozzle-based fluidic pick-and-place bioassembly technique designed to manipulate spheroids, organoids, or tissue fragments with high positional accuracy and minimal mechanical damage. AAB utilizes controlled suction to secure individual cellular aggregates, maintaining negative pressure until the building block is precisely positioned and subsequently released. Instead of extruding a continuous bioink, the system uses negative pressure to aspirate discrete spheroids into a capillary or pipette nozzle and then deposits them at predetermined coordinates by equilibrating the pressure [218].

**Figure 8:**
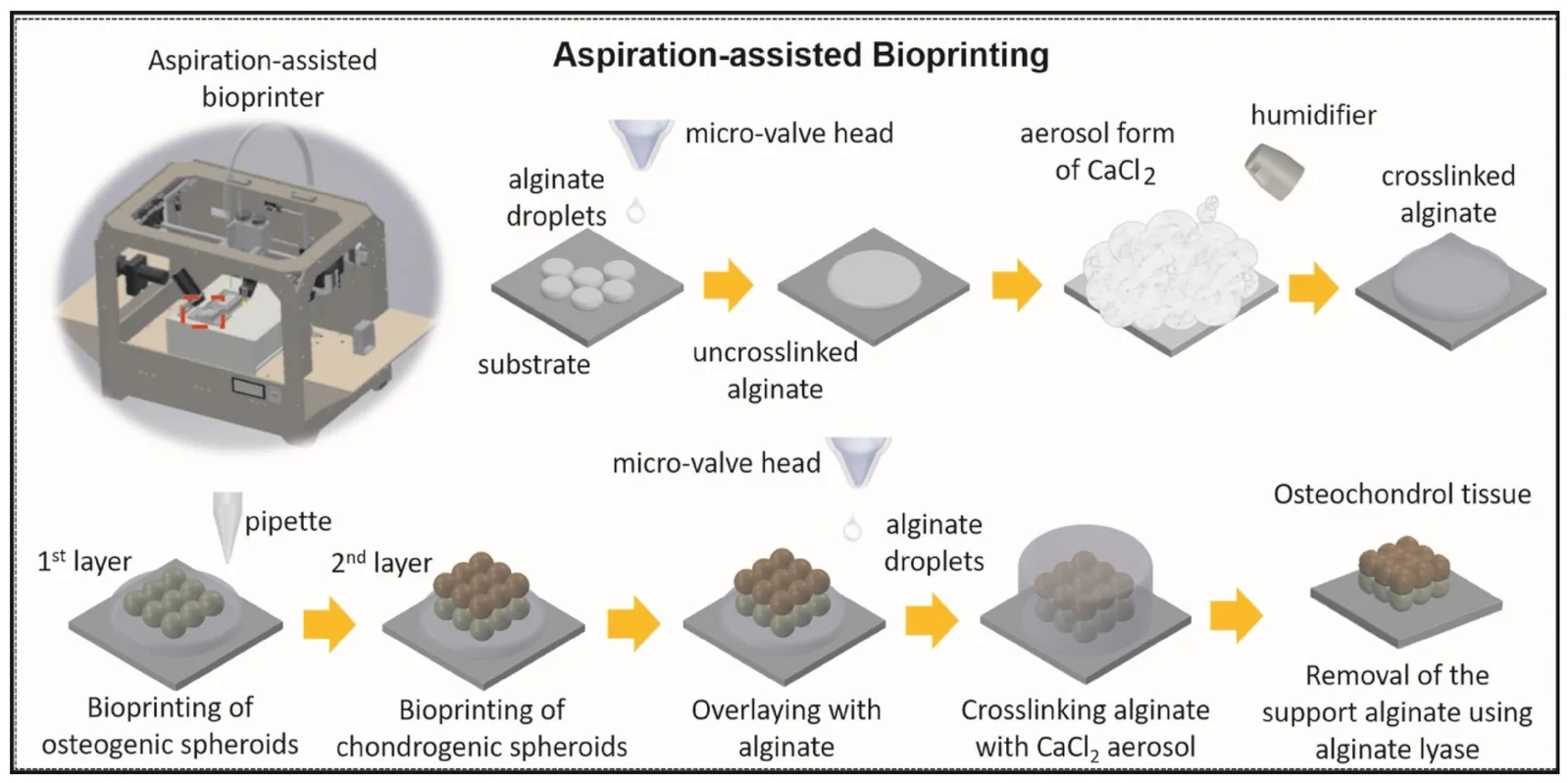
A schematic illustration showing the aspiration-assisted bioprinting (AAB) of the OC interface with chondrogenic and osteogenic zones. Reproduced from [219], licensed under CC BY 4.0.

The primary advantage of AAB is its ability to handle discrete biological building blocks while preserving their viability, structural architecture, and cell–cell contacts. Because the spheroids are not forced through a narrow nozzle as a continuous slurry, shear stress is significantly reduced compared with extrusion-based systems. The method also allows precise patterning of heterogeneous spheroids, enabling the fabrication of constructs with controlled spatial distribution of distinct cell types. For example, endothelial, stromal, epithelial, chondrogenic, osteogenic, or tumor spheroids were arranged in defined configurations to reproduce tissue interfaces, vascular niches, or disease microenvironments. In regenerative medicine, AAB is utilized to construct modular tissues composed of spheroids with different lineage commitments, such as osteochondral interfaces, dermal-epidermal constructs, and cardiac or hepatic microtissues. Ayan et al. [219] demonstrated 3D bioprinting of a scaffold-free dual-layered osteochondral interface using AAB. Human adipose-derived stem cell spheroids were differentiated into osteogenic and chondrogenic lineages, then precisely layered onto a sacrificial alginate support. Zone-specific fusion and phenotypic maintenance confirmed successful biofabrication, offering great potential for regenerative medicine, drug screening, and osteoarthritis modeling. The development of methods for the deterministic positioning of living organisms is critical for advanced bioimaging, cybernetics, cryopreservation, and organism-integrated engineering (Figure 8). Conventional approaches are limited by the need to continuously track randomly distributed organisms and transfer them without inducing mechanical damage. To address this challenge, Han and colleagues developed an aspiration-assisted adaptive printing system that tracks, harvests, and relocates live, motile organisms via a pick-and-place mechanism guided by real-time computer vision. This system successfully handles single static organisms, multi-organism droplets, and actively moving specimens. The utility of the platform is demonstrated through diverse proof-of-concept applications, including the deposition of vitrification-ready organisms in cryoprotectant droplets, automated sorting of live versus dead specimens, conformal printing on complex curved topologies, and the assembly of organism-powered displays. Ultimately, these adaptive strategies establish a foundation for fully autonomous biomanufacturing modalities to evaluate, sort, and assemble living organisms for next-generation multi-organism technologies [220].

Despite these advantages, AAB remains slower than continuous extrusion printing because aggregates are precisely controlled with aspiration pressure, one at a time or in small groups to avoid spheroid deformation. To mitigate this throughput limitation, multi-nozzle, digitally controlled aspiration arrays have been developed to pick multiple spheroids simultaneously and deposit them rapidly onto a substrate. In addition, temporary support matrices, such as agarose molds or sacrificial hydrogels, are required to provide structural stability until spheroid fusion yields sufficient cohesive strength [221].

#### 4.1.3. Magnetic Bioprinting and Field-Directed Tissue Assembly

Magnetic levitation bioassembly, a key modality in magnetic tissue engineering, utilizes magnetic forces to organize cells, spheroids, or cell-laden microgels into 3D structures without relying on solid molds or permanent scaffolds. In these systems, cells are typically labeled with magnetic nanoparticles or nanoparticle assemblies, after which an external magnetic field is applied to levitate, concentrate, or pattern the cells into defined 3D configurations. Haisler et al. [208] reported a magnetic levitation-based 3D culture technique where cells were functionalized with magnetic nanoparticles; upon resuspension in medium, an external magnetic field lifted and concentrated the cells at the air-liquid interface, driving rapid aggregation into 3D cultures. This approach laid the groundwork for high-throughput magnetic 3D bioprinting. By circumventing the need for exogenous synthetic protein substrates, this method facilitates the deposition of an endogenous ECM, offers precise spatial control, and enables the rapid assembly of dense, tissue-like constructs under controlled magnetic guidance.

The primary advantage of magnetic bioassembly is its ability to rapidly generate cell-dense constructs and manipulate living aggregates without direct mechanical contact. For instance, superparamagnetic iron oxide nanoparticles (SPIONs) magnetize only in the presence of an external field and demagnetize upon its removal. This allows them to serve as dynamic patterning agents, enabling spheroids and microtissues to be reversibly attached, concentrated, or released on demand. Consequently, directed magnetic fields can guide tissue morphogenesis and promote cell–cell contact to form rings, sheets, spheroids, tubes, and complex patterned microtissues. Demonstrating this capability, Souza et al. [222] developed the first 3D in vitro model of human uterine contractility utilizing high-throughput magnetic 3D bioprinting. Patient-derived and commercial human myometrial smooth muscle cells (SMC-A and SMC-B) were patterned into rings within 384-well plates. These bioprinted constructs exhibited origin-specific contraction profiles and differential sensitivity to clinical inhibitors (such as indomethacin and nifedipine), offering a scalable platform for evaluating parturition physiology. Similarly, Tseng et al. [223] introduced a high-throughput vasoactivity assay based on magnetic 3D bioprinting to replace low-throughput wire myography. Vascular smooth muscle cells were printed into functional 3D rings mimicking native blood vessels. Mobile-device imaging was then used to track pharmacological contraction and dilation, yielding results that correlated closely with in vivo outcomes and establishing a cost-effective platform for vasoactive drug screening. In another application, Ahmed et al. [224] developed a decellularized ECM (dECM) platform from porcine submandibular glands (SMG) using magnetic bioassembly on porous polycarbonate track-etched (PCTE) membranes. Perfusions with 0.1% and 1% sodium dodecyl sulfate efficiently cleared DNA (<50 ng/mg) while preserving key matrix components such as sulfated glycosaminoglycans, collagens, and elastin, as well as the native microarchitecture. The resulting dECM scaffolds significantly enhanced primary cell viability, proliferation, and acinar epithelial differentiation, establishing a robust strategy for salivary gland organogenesis [224].

Beyond these specific examples, magnetic systems are widely used to engineer tumor models, vascular networks, and hepatic or cardiac microtissues for high-throughput drug screening. Yoon et al. [225] developed a droplet-based microfluidic strategy encapsulating cells and magnetic nanoparticles within alginate beads to mimic multicellular tumor spheroid functions, demonstrating the utility of magnetic manipulation in cancer modeling. In regenerative medicine, magnetic tissue engineering holds significant potential for assembling vascularized microtissues, cartilage-like constructs, cardiac patches, and stem cell spheroids with enhanced intercellular signaling. Additionally, magnetic actuation has been used to mechanically stimulate constructs during maturation or to position cell aggregates precisely within irregular anatomical defects. The ability to manipulate constructs remotely is particularly valuable for minimally invasive or mold-free tissue assembly. However, translating these magnetic bioassembly strategies requires rigorous evaluation of nanoparticle biocompatibility, optimal dose, intracellular localization, long-term retention, and possible interference with cell phenotype or lineage differentiation.

### 4.2. Organoid-Based Bioprinting and Hierarchical Tissue Assembly

Organoid-based bioprinting is an emerging strategy of scaffold-free biofabrication in which self-organized 3D organoids are utilized as biological building blocks for the construction of larger and more structurally complex tissues. Organoids are multicellular microtissues derived from stem cells, progenitor cells, or primary tissue cells that possess the intrinsic capacity to recapitulate key aspects of native organ architecture, cellular heterogeneity, and functional specialization. Bioprinted organoids involve either the printing of stem cells into a tissue geometry that subsequently differentiates into organoids, or the assembly of pre-induced organoids within bioinks to replicate a native organ [226,227]. Compared with conventional spheroids, organoids exhibit a higher degree of tissue-specific organization, including multiple interacting cell populations and spatially defined microanatomical features that more closely resemble in vivo conditions.

Integrating organoids into bioprinting strategies requires overcoming a fundamental trade-off inherent to these structures. Unlike spheroids, which are relatively homogeneous and mechanically tolerant, organoids possess conserved, biologically functional internal cytoarchitectures that are vulnerable to disruption [228]. Bioprinting achieves precise spatial and temporal control of cell–cell interactions; however, conventional methods lack the optimization required for organoids due to their susceptibility to plastic deformation. This structural constraint has directly motivated the development of low-impact, contact-minimized organoid-handling technologies, in contrast to conventional extrusion-based bioprinting approaches that risk shear-induced disruption of internal organoid structure [229].

To address this limitation, the Spatially Patterned Organoid Transfer (SPOT) platform was developed. SPOT consists of an iron-oxide nanoparticle-laden hydrogel and a magnetized 3D printer that enables the controlled lifting, transport, and deposition of organoids. Cellulose nanofibers were identified as both an ideal biomaterial for encasing organoids with magnetic nanoparticles and a shear-thinning, self-healing support hydrogel to maintain spatial positioning during assembloid generation [230]. Using SPOT, the robust integration of hiPSC-derived ventral and dorsal forebrain organoids was achieved following controlled spatial positioning within and subsequent release from a cellulose nanofiber support scaffold. Over two weeks post-release, extensive migration of gamma-aminobutyric acid (GABA)ergic interneurons from the ventral region into the dorsal forebrain region of the assembloids was observed, with migratory cells exhibiting highly branched projections spanning a Z-depth as wide as 25 µm. This observation was the demonstration of spatial precision; the tangential migration of GABAergic interneurons from the ventral to the dorsal forebrain is a hallmark process of human corticogenesis. Consequently, SPOT-assembled organoids were shown to recapitulate developmental cell-migration behavior rather than static co-localization. SPOT was further utilized to construct disease-relevant cancer assembloids. By embedding hiPSC-derived cortical organoids and patient-derived glioma organoids in the iron nanoparticle-laden scaffold, the magnetized 3D printer generated precisely arranged neural assembloids and glioma-neural organoid complexes with controlled spatial patterning [230].

Complementary pick-and-place strategies have extended organoid assembly toward greater scalability. The Bio-Pick, Place, and Perfuse (Bio-P3) bioprinting method was developed to form scalable tissue constructs using large spheroids and organoids, thereby addressing the throughput limitations associated with single-unit manual placement [231]. A notable convergence of organoid bioprinting and vascularization strategy is found in cardiac tissue engineering. An embedded bioprinting technique termed sacrificial writing into functional tissue (SWIFT) was used to construct highly perfusable cardiac tissue by directly printing vascular channels into a living 3D matrix of densely packed hiPSC-derived cardiac spheroids. This approach achieved a synchronously beating tissue through spheroid fusion to form perfused and vascularized cardiac tissue [232]. Cardiac organoids bioprinted using this perfusable strategy spontaneously synchronized their contractions after 7 days of culture, exhibiting rhythmic and rapid calcium wave propagation. This method utilizes bioactive matrices to bioprint personalized organoids with embedded vascular channels, yielding biological functions that closely resemble those of human tissue. The emergence of spontaneous, synchronized calcium wave propagation across a fused multi-organoid construct serves as a robust functional validation; it demonstrates that hierarchical organoid assembly generates a larger tissue mass with electrophysiological coordination across the fused construct that was entirely absent in any single constituent organoid prior to assembly [232].

## 5. Conclusions and Future Perspectives

Three-dimensional (3D) bioprinting has rapidly evolved from a novel biofabrication tool into an indispensable cornerstone of tissue engineering and regenerative medicine. Innovations and advancements in this field have significantly expanded biofabrication capabilities, progressing beyond basic architectural scaffolding toward the precise replication of complex, multicellular microenvironments across diverse tissue types. The development of advanced, multi-component bioinks, incorporating highly functionalized hydrogels and tissue-specific decellularized extracellular matrices (dECMs), continues to progress, exhibiting enhanced material printability and biological functionality. Furthermore, the integration of emerging high-resolution modalities, such as volumetric bioprinting, support-bath-assisted embedded printing, and scaffold-free spheroid/organoid-based bioassembly, has enabled macroscopic fabrication of structurally robust and hierarchically vascularized constructs. The integration of these advanced technologies and innovative materials has successfully driven the engineering of diverse biomimetic tissue models, ranging from skin and cartilage to intricate vascular networks and other functional tissue structures.

Despite these advancements, several technical and translational hurdles need to be resolved to facilitate the translation of 3D-bioprinted constructs into clinical applications. A principal challenge lies in engineering multi-scale vascular architectures, particularly in replicating the microcapillary networks necessary to sustain metabolic diffusion and long-term cell survival within large-scale organ models, which remains technically elusive. Additionally, optimizing in situ crosslinking kinetics and mitigating shear-induced mechanical degradation during high-resolution extrusion are critical to preserving long-term cell viability and lineage-specific phenotypic stability. These biofabrication challenges are exacerbated by manufacturing and regulatory barriers, bioink batch variability, and the logistics of clinical-grade cell expansion, which complicate Good Manufacturing Practice (GMP) compliance. Conventional sterilization methods (autoclaving, gamma irradiation, ethylene oxide) degrade proteins and compromise cell viability; it is required to use low-temperature alternatives such as supercritical CO_2_ or cold plasma treatment. Reproducibility further demands a shift from destructive quality control (QC) toward non-destructive, in-line monitoring, for example, optical coherence tomography, alongside robust cryopreservation or vitrification strategies to prevent metabolic decline during storage and transit. Regulatory ambiguity persists as well, since agencies such as the U.S. Food and Drug Administration (FDA) frequently classify bioprinted constructs as “Combination Products” or “Advanced Therapy Medicinal Products (ATMPs), prolonging approval timelines. Ultimately, industrial-scale translation requires transitioning from slow, single-nozzle extrusion toward high-throughput modalities such as volumetric bioprinting to achieve economic viability.

Moreover, next-generation 3D bioprinting demands a synergy between multi-disciplinary technologies such as machine learning and artificial intelligence. Integrating these computational architectures into the fabrication process will enable real-time defect monitoring, the autonomous optimization of processing parameters, and the predictive modeling of post-print tissue maturation. Additionally, the integration of 4D bioprinting, wherein printed scaffolds retain the capacity to autonomously modify their configuration, structural hierarchy, or functional properties in response to physiological stimuli, offers significant potential for the engineering of responsive, biomimetic tissue grafts. Ultimately, overcoming existing rheological and vascularization limitations through these advanced intelligent, adaptive engineering strategies will expedite the clinical transition of biofabrication by 3D bioprinting from benchtop laboratory-scale modalities to customizable, patient-specific regenerative therapeutics.

## Figures and Tables

**Table 1:** Classification, operational parameters, advantages, and limitations of Scaffold-based 3D bioprinting modalities.

3D Bioprinting Modality	Resolution	Bioink Viscosity	Cell Viability	Advantages	Limitations
Material extrusion bioprinting	≈100–1000 µm	≈30–6 × 10^7^ mPa·s	40–90%	Wide material/viscosity range; high cell density; low cost	Lower resolution; shear-induced cell damage at high viscosity
Droplet-based (inkjet/DoD) bioprinting	≈10–80 µm	<≈30 mPa·s	74–85% (up to >90% optimized)	High speed; fine spatial control; low cost	Restricted to low-viscosity/low cell-density inks; nozzle clogging
Laser-assisted bioprinting	≈10–50 µm	≤1–300 mPa·s	>90%	Nozzle-free (no clogging/shear); high viability and precision	Low throughput; costly setup; ribbon-coating step needed
Light-based bioprinting	≈2–50 µm	Photocurable resin-dependent	75–95%	High resolution; fast layer-free (volumetric) options	Photoinitiator/UV cytotoxicity; limited to photocrosslinkable inks

**Table 2:** Material extrusion-based 3D bioprinting for bone tissue engineering applications.

Bioink/Biomaterial	Type of 3D (bio)Printing	Target Tissue	Applications	References
Alginate/PVA/silk fibroin	Extrusion-based (pneumatic/piston)	Bone (maxillofacial)	Scaffold supporting MC3T3-E1 osteoblast proliferation with enhanced ALP activity and calcium deposition	[56]
Chitosan/silk fibroin/cellulose nanoparticles (CS/SF/CNP)	Extrusion-based	Bone (immuno-modulatory)	Promoted macrophage polarization; upregulated osteogenic genes via the MAPK pathway in RAW 264.7 macrophages	[57]
Methacrylated silk fibroin/methacrylated hollow mesoporous silica microcapsules (HMSC-MA) with ciprofloxacin	Micro-extrusion-based	Bone (infection and defect)	Aerogel scaffold with controlled drug delivery; promoted cellular ingrowth, proliferation, and osteoblastic differentiation	[58]
High-density insoluble collagen fibrils/fibers dispersion (pH 4.0) (EDC crosslinking)	Extrusion-based 3D plotting	Bone/Adipose	High-fidelity scaffolds with hierarchical porosity; hMSC cytocompatibility with osteogenic and adipogenic differentiation potential	[59]
Collagen/dECM/silk fibroin (CES) composite	Low-temperature extrusion-based	Bone	Enhanced structural fidelity, mechanical strength, and MC3T3-E1 osteogenic activity	[60]
Silk fibroin/collagen/hydroxyapatite (SF/COL/HA)	Low-temperature extrusion-based	Bone	Enhanced compressive modulus and osteogenic differentiation	[61]
Collagen/PVA/propranolol/hydroxyapatite (CPPH)	Filament-free extrusion	Bone (traumatic defect)	Sustained local β-blocker delivery; enhanced bone formation and BMSC migration	[62]
Collagen/tannic acid with hASCs	Extrusion-based	Bone/General tissue	ECM-mimetic bioink with high cell metabolic activity	[60,63]
Collagen (5 wt%) with genipin (crosslinker) and osteoblast-like MG63/hASCs	Extrusion-based	Bone (hard tissue)	Elevated ALP, BMP-2, RUNX2, Col-I and OCN expression	[64]
PCL/silk fibroin microfibers	FFF-type extrusion	Bone (calvarial defect)	Enhanced hGMSC osteogenesis and bone regeneration in the rabbit model	[65,66]
Silk scaffold with HDAC2/3 inhibitor MI192	Extrusion-based	Bone (epigenetic)	Epigenetic reprogramming promoted bone-like tissue formation	[67]
PCL/Bombyx mori silk microparticles (PCL/SMP composites)	FFF-type extrusion	Bone	Improved compressive modulus and hADMSC activity	[68]
SF/gelatin/hyaluronic acid/tricalcium phosphate with PRP	Extrusion-based	Bone	Enhanced osteogenic markers of hADMSC	[69]
SF/gelatin/propanediol composite	Extrusion-based	Bone	Enhanced osteogenic gene expression through Smad1/5/8-RUNX2 signaling in MC3T3-E1	[70]
Silk fibroin/hydroxyapatite (SF/HA) nanocomposite in sodium alginate	Extrusion-based	Bone	Porous scaffold with apatite formation and hBMSC differentiation	[71]
Silica–silk fibroin gel with SH-CM-RGD peptide	Micro-extrusion and freeze-drying	Bone (infection-resistant)	Antibacterial porous aerogel scaffold with osteoconductivity	[72]
α-tricalcium phosphate/alginate with MC3T3-E1 (core/shell)	Extrusion (screw-rotating)-based	Bone	Core–shell construct with enhanced compressive strength	[73]
α-tricalcium phosphate/collagen composite with MC3T3-E1	Robotic extrusion-based	Bone	Multilayered bioceramic scaffold with mineralization enhancement	[74]
Alginate/gelatin with SaOS-2 overlaid with agarose/polyP·Ca^2+^	Extrusion-based	Bone	Enhanced proliferation and mineralization	[75]
Collagen/hydroxyapatite (CHA) composite	Low-temperature freeform fabrication (LDM)	Bone	Supported BMSC proliferation and improved bone repair in the rabbit femoral condyle defect model	[76]
Type I collagen/nanohydroxyapatite or deproteinized bovine bone (DBB)	Low-temperature freeform fabrication (LDM)	Bone	Osteoconductive bioinks supporting hBMSC differentiation	[77]
Gelatin/nanohydroxyapatite	Extrusion-based	Bone	Enhanced scaffold stiffness and osteoconductivity in vivo	[78]
Silk fibroin/gelatin/bioactive glass (Sr-doped) with TVA-BMSCs	Direct ink writing (DIW)	Bone	Enhanced osteogenic differentiation and signaling	[79]
Mesoporous bioactive glass/silk fibroin	Integrated extrusion printing	Bone	Osteogenicity of hBMSC-laden scaffolds in nude mice	[80]
Bioactive glass/graphene oxide composite	Direct ink writing (DIW)	Bone	TPMS architecture with improved mechanics and bioactivity	[81]
Tyramine-modified gelatin/silk fibroin/copper-doped bioactive glass (Cu-BG) with hUVECs	Direct ink writing (DIW)	Bone (angiogenic and osteogenic)	HIF-1α-mediated angiogenesis and bone repair	[82]
Calcium-deficient hydroxyapatite (CDHA)/alginate with MC3T3-E1 cells (core–shell)	Combined 3D printing and cell printing	Bone	Core–shell scaffold with >90% cell viability	[73]
Fibrillated collagen/β-tricalcium phosphate with hASCs	Extrusion-based	Bone	Spontaneous osteogenic differentiation without supplements	[83]
Bioglass 45S5 in methacrylated collagen	Extrusion-based	Bone	Accelerated mineral deposition and ALP activity by hMSCs	[84]
Silk fibroin/nanohydroxyapatite composite filament	Fused filament fabrication (FFF)	Bone (spinal fusion)	Cervical interbody fusion cage with reduced stress shielding	[85]
PLA/PCL/HA hybrid filament coated with silk fibroin	Fused filament fabrication (FFF)	Bone (fracture fixation)	Intramedullary nails with enhanced bone cell proliferation	[86]
PLA/hyaluronic acid/silk composite filament	Fused filament fabrication (FFF)	Bone (bone clip)	Patient-customized fracture fixation clips	[87]

**Table 3:** Material extrusion-based 3D bioprinting for cartilage and musculoskeletal (osteochondral) tissue engineering applications.

Bioink/Biomaterial	Type of 3D (bio)Printing	Target Tissue	Applications	References
Whey protein microgel-based granular hydrogels (WMGHs) with polyacrylamide	Embedded 3D Bioprinting	Cartilage	Patient-specific cartilage constructs with increased toughness	[88]
High-density type I collagen hydrogels with fibrochondrocytes	Extrusion-based	Meniscal cartilage	High geometric precision and mechanical strength, exhibiting high cell viability	[89]
Gelatin methacrylate (GelMA)	Extrusion-based	Hyaline/fibrocartilage	Superior printability and fidelity; formation of fibrocartilage-like tissue containing both type I and II collagens	[90]
Sodium alginate–nanocellulose composite	Extrusion-based	Cartilage	Enhanced mechanics and print resolution	[91]
Sodium alginate/collagen (SA/COL) with chondrocytes	Extrusion-based	Cartilage	Preserved chondrocytic phenotype; promoted the expression of cartilage-specific markers	[92]
CB-HA/DAH-HA supramolecular hydrogel	Extrusion-based	Cartilage	Supported hMSC chondrogenic differentiation	[93]
Alginate, Type I collagen, fibrin in thermoreversible gelatin microparticle bath	FRESH bioprinting	Cartilage	High-fidelity printing of complex geometries	[94]
Type I collagen with human nasal chondrocytes and BM-MSCs	Extrusion-based	Nasal cartilage	Homogeneous cell distribution, vascularized, and calcified in vivo	[95]
Type I collagen with human nasoseptal chondrocytes (hNCs)	FRESH bioprinting	Nasoseptal cartilage	Stable cell-laden structures	[96]
Type I collagen with hNCs	FRESH bioprinting	Nasal reconstruction	Customized cartilage grafts	[97]
MonoCB [6]/DAH-HA supramolecular hydrogel with atelocollagen containing hTMSCs	Extrusion-based	Osteochondral	Multilayered construct for osteochondral regeneration in rabbit knee defects	[98]

**Table 4:** Material extrusion-based 3D bioprinting for skin tissue engineering applications.

Bioink/Biomaterial	Type of 3D (bio)Printing	Target Tissue	Application	References
Gelatin/alginate with dermal fibroblasts	Extrusion-based	Skin	Burn wound dressing; improved wound closure	[99]
Collagen with human iPSC-derived fibroblasts and keratinocytes	Extrusion-based	Skin	Full-thickness artificial skin model	[100]
Type I collagen with fibroblasts and keratinocytes	Multilayer extrusion-based	Skin	Vascularized skin graft with microvascular networks	[101]
Silk fibroin hydrogel	Extrusion-based	Skin	Bioactive burn wound dressing	[102]
Silk fibroin/PVA/methylcellulose	Extrusion-based	Skin	Ultra-stretchable cytocompatible hydrogel with human keratinocytes	[103]
Sodium alginate/carboxylated cellulose nanocrystals/xanthan gum with fibroblasts	Extrusion-based (Direct ink writing)	Skin	Enhanced fibroblast adhesion and proliferation	[104]
Alginate-gelatin (core) with MSCs/methylcellulose (shell)	Coaxial extrusion bioprinting	Skin	Improved shape fidelity and MSC viability	[105]
Methacrylated κ-carrageenan with NIH/3T3 fibroblasts	Coaxial extrusion bioprinting	Skin	Stable dual-crosslinked hydrogels exhibiting high cell viability	[106]
GelMA/collagen-doped with tyrosinase with skin cells	Extrusion-based	Skin	Living skin tissue constructs	[107]
Collagen hydrogel precursor with fibroblasts and keratinocytes	Freeform fabrication (FFF)	Skin	Customized bilayer (dermal-epidermal) skin grafts	[108]
PLA scaffold with mechanobactericidal nanotopography	Extrusion-based printing with surface modification	Skin/Bone	Antimicrobial scaffold with osteogenic support	[109,110]
CHMA/PVA hydrogel containing BMSCs	Granular bioink bioprinting	Skin/Organoid	Self-healing bioink; spheroid formation	[111]

**Table 5:** Droplet-based bioprinting (DBB) for various tissue engineering applications.

Bioink/Biomaterial	Type of DBB	Target Tissue	Applications	References
Agarose/type I collagen blend hydrogel-laden with mesenchymal stem cells	Inkjet/DoD 3D bioprinting	Bone	Promoted osteogenic differentiation; BMP-2/TGF-β incorporation, and endothelial co-culture for vascularization	[150]
Type I collagen/calcium phosphate composite	Inkjet-based (low-temperature)	Bone	Constructs loaded with osteoinductive factors enhanced bone healing in critical-size defect models	[151]
Multi-material bioink (cell-laden hydrogel constructs)	Microvalve bioprinting	General/multi-material tissue constructs	Maintenance of post-printing cell viability using L929 mouse fibroblasts and human MSCs across multi-component architectures	[152]
Low-viscosity type I collagen bioink (with vapor-phase ammonia crosslinking for in situ gelation)	DoD bioprinting	Skin	3D bioprinting of structurally stable collagen constructs for dermal tissue engineering with preserved cell viability	[153]
Silk fibroin/nano or micro bioglass particles	Indirect DoD inkjet 3D printing + freeze-drying	Bone	Improved hBMSC adhesion and ALP activity	[154]

**Table 6:** Laser-mediated and light-assisted 3D bioprinting for various tissue engineering applications.

Bioink/Biomaterial	Crosslinking	Photoinitiator, Source, and Wavelength	Type of 3D Bioprinting	Tissue Engineering	References
Methacrylated silk fibroin	Free-radical photopolymerization of pendant methacrylate groups	DLP (405 nm, visible-blue light); photoinitiator (LAP)	DLP (vat photopolymerization)	Bone (pre-osteoblast-laden scaffolds)	[171]
Silk fibroin methacrylated–bacterial nanocellulose composite	Photocrosslinking of methacrylate groups	DLP (405 nm, visible-blue light); photoinitiator (LAP)	DLP (vat photopolymerization)	Bone (MC3T3-E1 pre-osteoblast-laden scaffolds)	[172]
Methacrylated κ-carrageenan/bioactive silica nanoparticles	Photocrosslinking of methacrylate groups	DLP (405 nm, visible-blue light); photoinitiator (LAP)	DLP (vat photopolymerization)	Bone (MC3T3-E1 pre-osteoblast-laden scaffolds; in vivo Wistar rat)	[173]
PEG-based hydrogel bilayers (differing molecular weight)	UV photopolymerization with differential-strain self-folding	UV photolithography; photoinitiator (Irgacure 2959)	Photopatterning/self-folding (bio-origami)	Pancreatic islet model (insulin-secreting β-TC-6 cells); ductal carcinoma model	[174]
PEG-diacrylate (PEGDA)/methacrylated gelatin (methagel) bilayer	UV photopolymerization with differential-strain self-folding	UV photolithography; photoinitiator (Irgacure 2959)	Photopatterning/self-folding (bio-origami)	Breast tissue (mammary duct/acinus model; ductal carcinoma, MDA-MB-231 cells)	[175]
Glycidyl methacrylate–functionalized silk fibroin (Sil-MA)	Free-radical photopolymerization of methacrylate groups	DLP (365 nm, UV-LED); photoinitiator (LAP)	DLP (vat photopolymerization)	Cartilage/multi-organ-mimetic constructs (heart, vessel, brain, trachea, ear)	[176]
Glycidyl methacrylate-modified silk fibroin (Silk-GMA)	Photocrosslinking of methacrylate groups	DLP (365 nm, UV-LED); photoinitiator (LAP)	DLP (vat photopolymerization)	Cartilage (chondrocyte-laden; rabbit trachea model in vivo)	[177]
Type I collagen	Riboflavin-mediated radical photopolymerization	Riboflavin (photoinitiator/photosensitizer); UV irradiation (265 nm)	Direct UV photo-crosslinking (non-vat, applied to printed collagen)	Soft-tissue/cartilage-mimetic scaffolds	[178]
Type I collagen	Riboflavin-mediated, pH-dependent photocrosslinking	Riboflavin; blue light (400–500 nm)	Extrusion bioprinting and secondary photocrosslinking	Cartilage (bovine chondrocyte-laden)	[179]
Collagen-embedded dermal fibroblasts and keratinocytes	physical laser-induced ejection; collagen self-assembles thermally post-deposition	pulsed laser focused onto a metal (e.g., gold/titanium) absorbing layer that vaporizes to eject bioink	Laser-assisted bioprinting (LIFT)	Skin (dermal/epidermal bilayer)	[180,181,182,183]
Methacrylated κ-carrageenan (MA-κ-CA)	Photocrosslinking of methacrylate groups	DLP (405 nm, visible blue); photoinitiator (LAP)	DLP (vat photopolymerization)	Skin (NIH/3T3 fibroblast-laden)	[184]
Gelatin methacrylamide (GelMA)	Photocrosslinking with differential crosslink density driving curling/self-rolling	Irgacure; UV light	Photopatterning/self-folding (UV-driven shape-morphing)	Vascular (microvascular tubules, HUVEC-lined; in vivo rat flap model)	[185]

## Data Availability

No new data were created or analyzed in this study. Data sharing is not applicable to this article.
